# CXCL5 suppression recovers neovascularization and accelerates wound healing in diabetes mellitus

**DOI:** 10.1186/s12933-023-01900-w

**Published:** 2023-07-07

**Authors:** Ching Chen, Liang-Yu Lin, Jaw-Wen Chen, Ting-Ting Chang

**Affiliations:** 1 Department and Institute of Pharmacology, National Yang Ming Chiao Tung University, Taipei, Taiwan; 2 School of Medicine, National Yang Ming Chiao Tung University, Taipei, Taiwan; 3 Division of Endocrinology and Metabolism, Department of Medicine, Taipei Veterans General Hospital, Taipei, Taiwan; 4 Healthcare and Services Center, Taipei Veterans General Hospital, Taipei, Taiwan; 5 Division of Cardiology, Department of Medicine, Taipei Medical University Hospital, Taipei, Taiwan; 6 Cardiovascular Research Center, National Yang Ming Chiao Tung University, Taipei, Taiwan; 7 Cardiovascular Research Center, Taipei Medical University, Taipei, Taiwan; 8 Biomedical Industry Ph.D. Program, National Yang Ming Chiao Tung University, Taipei, Taiwan

**Keywords:** CXCL5, Diabetes mellitus, Neovascularization, Wound healing, CXCR2

## Abstract

**Background:**

Higher chemokine C-X-C motif ligand 5 (CXCL5) level was observed in type 2 diabetes mellitus (DM) patients; however, its role in diabetic vasculopathy was not clarified. This study aimed to explore the impacts and mechanistic insights of CXCL5 in neovasculogenesis and wound healing in DM.

**Methods:**

Endothelial progenitor cells (EPCs) and human aortic endothelial cells (HAECs) were used in vitro. Streptozotocin-induced diabetic mice and Lepr^db^/JNarl mice were used as type 1 and type 2 DM models. Moreover, CXCL5 knockout mice were used to generate diabetic mice. Hindlimb ischemia surgery, aortic ring assays, matrigel plug assay, and wound healing assay were conducted.

**Results:**

CXCL5 concentrations were increased in plasma and EPCs culture medium from type 2 DM patients. CXCL5 neutralizing antibody upregulated vascular endothelial growth factor (VEGF)/stromal cell-derived factor-1 (SDF-1) and promoted cell function in EPCs from type 2 DM patients and high glucose-treated EPCs from non-DM subjects as well as HAECs. CXCL5 directly up-regulated interleukin (IL)-1β/IL-6/tumor necrosis factor-α and down-regulated VEGF/SDF-1 via ERK/p65 activation through chemokine C-X-C motif receptor 2 (CXCR2). CXCL5 neutralizing antibody recovered the blood flow after hindlimb ischemia, increased circulating EPC number, and enhanced VEGF and SDF-1 expression in ischemic muscle. CXCL5 suppression promoted neovascularization and wound healing in different diabetic animal models. The above observation could also be seen in streptozotocin-induced CXCL5 knockout diabetic mice.

**Conclusions:**

CXCL5 suppression could improve neovascularization and wound healing through CXCR2 in DM. CXCL5 may be regarded as a potential therapeutic target for vascular complications of DM.

**Supplementary Information:**

The online version contains supplementary material available at 10.1186/s12933-023-01900-w.

## Background

Diabetes mellitus (DM) is a chronic metabolic disease. Patients suffer from insufficient insulin secretion or resistance to insulin, which causes hyperglycemia [1–3]. Hyperglycemia causes decreased proliferation of endothelial cell and lower endothelial progenitor cells (EPCs) recruitment, which results in dysfunctional angiogenesis. In fact, dysfunctional angiogenesis has been involved in vascular complications of DM. Peripheral artery disease (PAD) is one of the most serious complications [4, 5] and the main causes of death in patients with DM [6]. Furthermore, DM patients with PAD have impaired wound healing [7]. For treatment of large vessel occlusion in severe PAD, surgical revascularization is usually performed, but for extensive and/or small vessel ischemia (2–5 mm vessel diameter), angiogenic drug treatment and regeneration therapy are the major choice relied upon [8]. Neocapillary vessel formation and remodeled response are involved in recovered blood flow to ischemic tissue. Therefore, the therapeutic potential of emerging neovascularization strategy is urgently needed for diabetic vasculopathy.

Chemokine C-X-C motif ligand 5 (CXCL5), also known as epithelial neutrophil activating peptide, is a chemokine that is mainly implicated in the chemotaxis of inflammatory cells [9, 10]. CXCL5 promotes neutrophil migration and activates inflammatory response through chemokine C-X-C motif receptor 2 (CXCR2) [11]. CXCL5 level was increased in mouse models of DM and in clinical settings [12–16]. CXCL5 could induce insulin resistance by inhibiting the insulin signaling pathway in muscle, adipose tissue, and macrophages. Increased CXCL5 levels were accompanied with impaired islet function [17]. Inhibition of CXCL5 by neutralizing antibody could improve insulin sensitivity and glucose clearance in insulin-resistant-obese mice [18, 19]. Taken together, CXCL5 may be increased in DM and involved in the development and progression of vascular diseases.

Theoretically, CXCL5 might contribute to the vascular complications in diabetes, though the direct link and detailed mechanism were not yet established. Given the clinical significance of diabetic vasculopathy, this study aimed to investigate the potential impacts and mechanistic insights of CXCL5 in diabetic vasculopathy. Serial in vitro and in vivo experimental models were used to evaluate if direct inhibition by neutralizing antibody or deficiency of CXCL5 by genetic knockout could improve diabetes related vasculopathy. The results may support the potential role of CXCL5 as a novel therapeutic target for diabetic vasculopathy.

## Methods

### In vitro study

#### Cell culture

The blood sample was collected from the peripheral veins of patients with type 2 DM and non-DM subjects. Only stable type 2 DM patients without insulin treatment were enrolled. Patients with other significant systemic diseases, receiving major operation in the past 6 months, or currently under medical treatment for other diseases were excluded. Demographic and clinical data were obtained at enrollment. Informed consent was obtained from all individual participants included in the study. The human study was approved by the institute research committee and conformed with the Declaration of Helsinki. This study was approved by the Institutional Review Board of Taipei Veterans General Hospital (IRB-2018-01-001AC).

After blood was collected, the total mononuclear cells were separated by Histopaque-1077 (Sigma-Aldrich, 10771, Darmstadt, Germany) and centrifuged at 500×*g* at room temperature for 30 min. The mononuclear cells were cultured in endothelial cell basal medium (Lonza, CC-3156, Basel, Switzerland), with supplements including hydrocortisone, human fibroblast growth factors, vascular endothelial growth factor (VEGF), R3-insulin-like growth factor-1, ascorbic acid, human epidermal growth factor, gentamicin sulfate-amphotericin and 20% fetal bovine serum on fibronectin-coated 6-well plates. After 2–4 weeks culture, attached EPCs were in the shape of cobblestones, and this kind of shape is the typical monolayer growth pattern of mature endothelial cells. Human aortic endothelial cells (HAECs; ScienCell, Catalog #6100, Carlsbad, CA, USA) were cultured with endothelium cell medium containing VEGF and 1% penicillin/streptomycin (Sigma-Aldrich, P4333, Darmstadt, Germany), and cultured dishes were coated with fibronectin before use. To mimic the hyperglycemia in DM, we administered 25 mM high glucose (HG) for 2 days to EPCs from non-DM subjects or HAECs. Some cells were treated with CXCL5 monoclonal antibody (1 or 10 μg/mL; R&D Systems, MAB-254, Minneapolis, MN, USA) or recombinant human CXCL5 protein (1 or 10 ng/mL; R&D Systems, 254-XB, Minneapolis, MN, USA). Cells were grown in culture medium supplemented with fetal bovine serum (5% v/v final concentration) in an atmosphere of 95% air and 5% CO_2_ at 37 °C.

In another part of the study, the following inhibitors were used to explore the signaling pathways: an extracellular signal-regulated kinase (ERK) inhibitor (10 μM; U0126; Cayman Chemical Company, No. 70970, Ann Arbor, MI, USA) and a selective CXCR2 antagonist (10 or 100 nM; SB332235; Cayman Chemical Company, No. 32869, Ann Arbor, MI, USA). The above reagents were added 1 h before the administration of the recombinant human CXCL5 protein.

#### Concentration of CXCL5

The concentration of CXCL5 was measured using an ELISA kit (R&D Systems, DX000 and MX000, Minneapolis, MN, USA) according to the manufacturer’s instructions.

#### Migration assay

To evaluate the basal cell migration ability of cells, the cells (1 × 10^4^ cells) were seeded on the upper chamber of 24-well transwell plate with polycarbonate membrane. Then, cells migrated toward the lower chamber containing 600 μL cultured medium with FBS at 37 °C in 5% CO_2_. After 18 h, migrated cells were fixed in 4% paraformaldehyde and stained with hematoxylin solution. Images were captured by a high-power (100×) microscope.

#### Network formation assay

The cells (1 × 10^4^ cells) were seeded into ECMatrix gel (Invitrogen, Carlsbad, CA, USA) in 96-well plates in 100 μL cultured medium with 10% FBS for 16 h at 37 °C in 5% CO_2_. Images were captured by high-power (40×) microscope. The numbers of formed networks of cells were calculated using Image-Pro Plus (Media Cybernetics, Inc. Rockville, MD, USA).

#### Western blot

Total cell or tissue lysates were extracted using lysis buffer, and proteins were separated in 8–12% (v/v) SDS-PAGE gels. After electrophoresis (Bio-Rad Laboratories, Hercules, CA, USA), the proteins were transferred onto PVDF membranes (Millipore, Darmstadt, Germany), and the membranes were incubated with anti-VEGF (Santa Cruz Biotechnology, sc-152, Dallas, TX, USA), anti-stromal cell-derived factor (SDF)-1 (Cell Signaling Technology, 3530S, Boston, MA, USA), anti-interleukin (IL)-1β (Santa Cruz Biotechnology, sc-52012, Dallas, TX, USA), anti-IL-6 (Santa Cruz Biotechnology, sc-1265, Dallas, TX, USA), anti-tumor necrosis factor (TNF)-α (Santa Cruz Biotechnology, sc-52746, Dallas, TX, USA), anti-p-ERK (Cell Signaling, 9106S, Boston, MA, USA), anti-ERK (Cell Signaling, 9102S, Boston, MA, USA), anti-CXCR2 (Santa Cruz Biotechnology, sc-7304, Dallas, TX, USA), anti-CXCL5 (R&D, MAB254, Minneapolis, MN, USA), anti-p-p65 (Cell Signaling Technology, #3031S, Danvers, MA, USA), anti-p65 (BD, 0079008, East Rutherford, NJ), and anti-actin (Merck, 3423208, Darmstadt, Germany) at 4 overnight. After washing three times, the membranes were incubated with HRP-conjugated secondary antibodies (1:1000) for 1 h at room temperature. Finally, the membranes were visualized using the ECL kit.

#### Immunoprecipitation

HAECs were incubated with CXCL5 1 or 10 ng/mL recombinant protein (R&D, 254-XB, Minneapolis, MN, USA) for 30 min. Cells were chilled on ice and cell extracts prepared with lysis buffer (1% Triton X-100, 2.5 mM EDTA, 25 mM Tris–HCl, 150 mM NaCl, 5% glycerol, 1 mM PMSF, pH 7.4). Lysates were cleared by centrifugation at 13,000 rpm for 30 min and incubated with anti-CXCR2 (Santa Cruz Biotechnology, sc-7304, Dallas, TX, USA) attached to agarose beads overnight at 4 °C on a rocking. Beads were then collected by centrifugation at 10,000 rpm for 3 min at 4 °C, extensively washed in lysis buffer. The proteins were separated on a 12% SDS-polyacrylamide gel, transferred to a PVDF membrane, and analyzed by immunoblotting with the corresponding antibodies.

### In vivo study

#### Animal model of DM

Etiologically distinct pre-clinical models were utilized independently. Six-week-old male FVB/NCrlBltw mice were purchased from BioLASCO (Taipei, Taiwan). Mice were injected intraperitoneally with 40 mg/kg streptozotocin (STZ; Sigma-Aldrich, S0130, Darmstadt, Germany) for 5 days. The experiment began when the mice’s blood sugar level was greater than 250 mg/dL. Six-week-old male BKS.Cg-m+/+Lepr^db^/JNarl mice were purchased from the National Laboratory Animal Center (Taipei, Taiwan). Mice were all acclimated for 2 weeks before experiments. For the treatment of hindlimb ischemia surgery, mice were injected intraperitoneally with CXCL5 monoclonal antibody (R&D, MAB433, Minneapolis, MN, USA) or IgG monoclonal antibody (R&D, MAB0061, Minneapolis, MN, USA) at 10 or 100 μg three times per week for a month.

Six-week-old male C57BL/6JNarl-Cxcl5^em1^ knockout mice were designed and purchased from the National Laboratory Animal Center (Taipei, Taiwan). CXCL5 knockout (CXCL5KO) mice were produced with a C57BL/6JNarl genetic background using the CRISPR/Cas9 system. Taken together, fertilized embryos of C57BL/6JNarl mice were microinjected with sgRNAs and Cas9 mRNA. For phenotypic analyses in parallel with age- and sex-matched wild-type (WT) littermates, homozygous knockout mice were produced from a heterozygous intercross. All mice were genotyped using PCR with specific primers (forward, 5′-TCTTAAAGGTTGAGCCATCTCCC-3′ and reverse, 5′-CCCATTATGATCTAAATCCCCACC-3′). Mice were raised under specific pathogen-free conditions and kept in micro isolator cages with 12:12-h light/dark cycles and free access to water and standard mouse chow. Mice were injected intraperitoneally with 40 mg/kg STZ for 5 days. The experiment began when their blood sugar level was greater than 250 mg/dL. All animal experiments were approved by the Institutional Animal Care and Use Committee (IACUC) of National Yang Ming Chiao Tung University (IACUC No. 1091104).

#### Hindlimb ischemia model

The unilateral hindlimb ischemia surgery has been used in our previous publication [20]. To be specific, mice were anesthetized by inhalation of 3% isoflurane and exposed for 10 min once a week. Mice were then shaved, and the surgical site was cleaned with 70% ethanol. The femoral artery and vein were separated from the femoral nerve, and the proximal and distal portions of the right femoral artery and the distal portion of the right saphenous artery were ligated. Then, the arteries and all side branches were dissected free and excised. The skin was closed with a noncontinuous suture. Hindlimb ischemia blood flow was analyzed by laser Doppler perfusion imaging (Moor Instruments Limited, Devon, UK) at days 0, 7, 14, 21, and 28 after surgery. The rate of reperfusion in the hindlimb was calculated as a ratio of blood flow in the ischemic limb compared with the nonischemic limb for each mouse. All mice were anesthetized via inhaled isoflurane and sacrificed through cardiac exsanguination 28 days after ischemic surgery. The gastrocnemius muscle from each leg was harvested for immunohistochemistry or protein expression analyses.

#### Flow cytometry

The peripheral blood mononuclear cells were suspended in saline and incubated with fluorescein isothiocyanate anti-mouse stem cells antigen (Sca)-1 (Invitrogen, 11-5981-82, Carlsbad, CA, USA) and phycoerythrin anti-mouse vascular endothelial growth factor receptor 2, also known as Flk-1 (Invitrogen, 12-5821-82, Carlsbad, CA, USA) at room temperature for 30 min. A BD FACScalibur flow cytometer (BD, East Rutherford, NJ) was used, and data were analyzed with FloJo (Treestar). Data are presented as % gated, relative to control group.

EPCs were harvested and washed prior to suspension in phosphate-buffered saline (PBS) for flow cytometry. To identify the characteristics of EPCs, VE-cadherin (Biolegend, 348505, San Diego, CA), CD31 (Biolegend, 303117, San Diego, CA), CD34 (BD, 555821, East Rutherford, NJ), KDR (R&D, FAP357, Minneapolis, MN, USA), CD133 (MACS, 130-111-756, Germany), CD3 (Biolegend, 300407, San Diego, CA), CD68 (Biolegend, 333805, San Diego, CA), CD86 (Biolegend, 374203, San Diego, CA), CD163 (Biolegend, 33605, San Diego, CA), and CD206 (Biolegend, 321123, San Diego, CA) antibodies were used. Cells were analyzed by BD FACScalibur flow cytometer (BD, East Rutherford, NJ), and data were analyzed with FloJo (Treestar). Data are presented as % gated.

#### Aortic ring assay

The aortic ring assay has been used in our earlier publication [21]. Aortic rings were then cut to 0.5 mm and embedded in 1 mg/mL type 1 rat tail collagen matrix (Millipore, 08115, Darmstadt, Germany) with incubation for 1 h at 37 °C. Aortic rings were cultured using endothelial cell growth basal medium-2 (Lonza, Bend, OR, USA) with supplements (hydrocortisone, human fibroblast growth factors, VEGF, R3-insulin-like growth factor-1, ascorbic acid, human epidermal growth factor, gentamicin sulfate-amphotericin) containing 2.5% fetal bovine serum (Gibco, Carlsbad, CA, USA), 50 U/mL penicillin, 0.5 mg/mL streptomycin (Sigma-Aldrich, P4333, Darmstadt, Germany), and 30 ng/mL VEGF (Peprotech, 100-20, Rocky Hill, CT, USA) in 24-well plates for 7 days. After culture, aortic rings were incubated with fluorescein isothiocyanate anti-lectin B4 (Sigma-Aldrich, L9006, Darmstadt, Germany) at 4 °C overnight. Images were captured using a fluorescent microscope (100×).

#### Matrigel plug neovascularization assay

The matrigel plug neovascularization assay has been used in our previous publication [22]. In detail, mice were anesthetized by inhalation of 3% isoflurane and exposed for 10 min in this assay. Mice were injected subcutaneously with growth factor reduced (GFR) basement membrane matrix (Corning® Matrigel, 356231, Glendale, AZ, USA) containing 30 ng/mL VEGF (Peprotech, 100-20, Rocky Hill, CT, USA) and 50 U/mL heparin (Sigma-Aldrich, H3393, Darmstadt, Germany). The gel formed a solid plug as it reached the body temperature. After 14 days, plugs were collected and homogenized with 500 μL cell lysis buffer and centrifuged at 6000×*g* at 4 °C for 60 min. A colorimetric assay (Sigma-Aldrich, MAK115, Darmstadt, Germany) was used to detect hemoglobin with a microplate reader at 400 nm wavelength. The plug was harvested for histological and immunohistochemistry analysis.

#### Wound healing assay

The wound healing assay has been used in our previous publication [22]. To be specific, mice were anesthetized by inhalation of 3% isoflurane and exposed for 3 min in this assay. The back skin was shaved and cleaned with antibacterial soap solution and 70% ethanol. Circular full-thickness excisional wounds of 3 mm diameter were generated by biopsy punch without muscle injury. Wounds were recorded using a digital camera (Nikon, Tokyo, Japan) at 0, 1, 3, 5 and 8 days after they were generated.

#### Histological and immunohistochemistry analysis

Tissue sample was fixed with 4% paraformaldehyde for 24 h, dehydrated in graded alcohols, and then embedded in paraffin wax. The tissues were sectioned into samples of 5 μm thickness. Sections were dried overnight and stained with hematoxylin and eosin and Masson’s trichrome stain for histological analysis. Paraffin wax-embedded tissues were sectioned at 5 μm thicknesses and rehydrated. Antigen retrieval was performed using 0.05 M sodium citrate buffer. Slides were then incubated at 4 °C overnight with the primary antibody to detect CD31 (Abcam, 124432, Waltham, MA, USA). The sample was washed with PBS solution and incubated with a secondary antibody (rabbit) for 2 h at room temperature. In Masson’s trichrome stain, Paraffin wax-embedded tissues were sectioned at 3 μm thicknesses, which were stained with Weigert’s iron hematoxylin working solution for 10 min. Then, the sections were stained with Biebrich scarlet-acid fuchsin solution and phosphomolybdic–phosphotungstic acid solution for 15 min. Finally, the sections were transferred to aniline blue solution for 10 min.

#### Mice blood glucose test

After 4 h of fasting, 1 μL of mouse blood was collected from the tail. An Abbott FreeStyle glucometer (Abbot-OPTIUM XCEED) was used following the instructions provided by the original manufacturer.

#### Statistical analysis

Given our data’s non-parametric nature, we utilized the median and interquartile for descriptive statistics and the Mann–Whitney U test to identify group differences. A *p*-value of less than 0.05 was deemed statistically significant.

## Results

### In vitro study

#### CXCL5 neutralizing antibody upregulated VEGF/SDF-1 expression and promoted angiogenesis in EPCs from type 2 DM patients

**Fig. 1:**
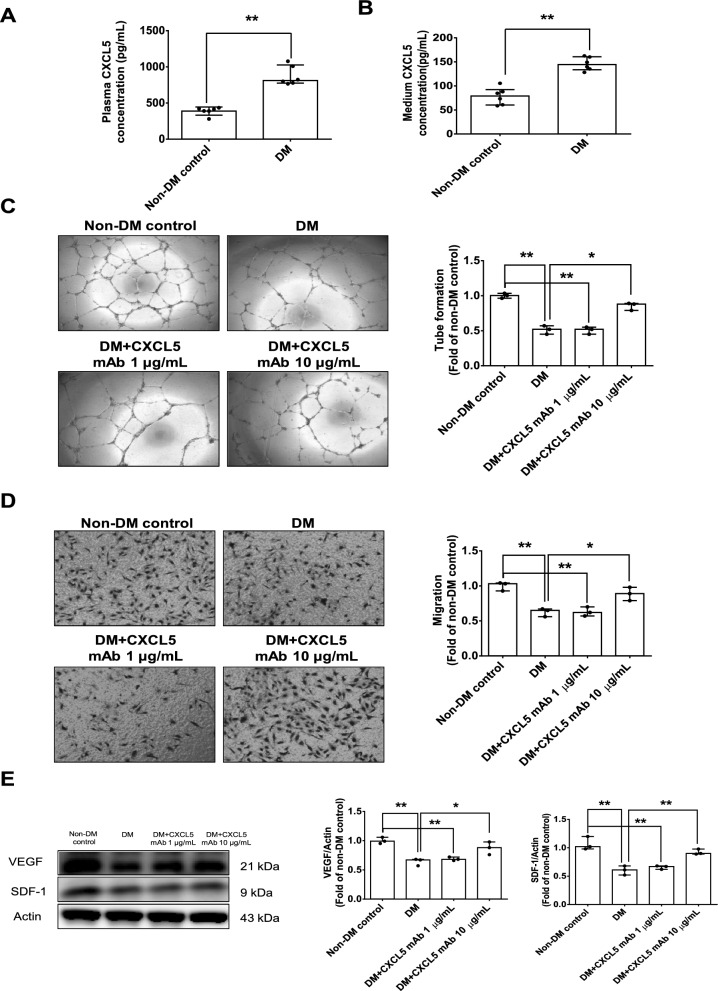
Treatment with CXCL5 neutralizing antibody upregulated VEGF/SDF-1 expression and promoted angiogenesis in late-EPCs from type 2 DM patients. Plasma levels of CXCL5 in type 2 DM patients and non-DM subjects (n = 6; **A**). EPCs medium levels of CXCL5 in type 2 DM patients and non-DM subjects (n = 6; **B**). The network formation and migration abilities were improved after the administration of CXCL5 mAb in EPCs from type 2 DM patients (n = 3; **C**, **D**). Western blotting and statistical analyses of VEGF and SDF-1 in EPCs from type 2 DM patients (n = 3; **E**). *CXCL5* Chemokine C-X-C motif ligand 5, *DM* diabetes mellitus, *EPC* endothelial progenitor cell, *mAb* monoclonal antibody, *SDF-1* stromal cell-derived factor 1, *VEGF* vascular endothelial growth factor. N represents cells cultured from n different individuals, and cells cultured from each individual were experimented for three independent experiments. The Mann–Whitney U test was used to determine statistically significant differences. *p < 0.05, **p < 0.01

The level of CXCL5 concentration was increased in plasma from patients with type 2 DM in comparison to non-DM subjects (Fig. 1A). The CXCL5 concentration in the culture medium of EPCs from type 2 DM patients was increased in comparison to non-DM subjects (Fig. 1B). Clinical characteristics of the study population was shown (Additional file 1: Table S1). The EPCs used in this study was positive for vascular endothelial cell markers such as VE-cadherin, CD31, CD34, KDR, CD133, and negative for macrophage markers such as CD3, CD68, CD86, CD163, and CD206 (Additional file 1: Fig. S1). Administration of CXCL5 neutralizing antibody in amounts of 10 µg/mL repaired the network formation and migration abilities of EPCs from type 2 DM patients (Fig. 1C, D). Treatment of CXCL5 neutralizing antibody at 10 µg/mL enhanced the protein expression of VEGF and SDF-1 in EPCs from type 2 DM patients (Fig. 1E). Therefore, these results indicated that CXCL5 neutralizing antibody could increase VEGF and SDF-1 protein expression to promote the migration and in vitro angiogenesis ability of EPCs from type 2 DM patients.

#### CXCL5 neutralizing antibody upregulated VEGF/SDF-1 expression and promoted angiogenesis in HG-stimulated EPCs from non-DM subjects and HAECs

**Fig. 2:**
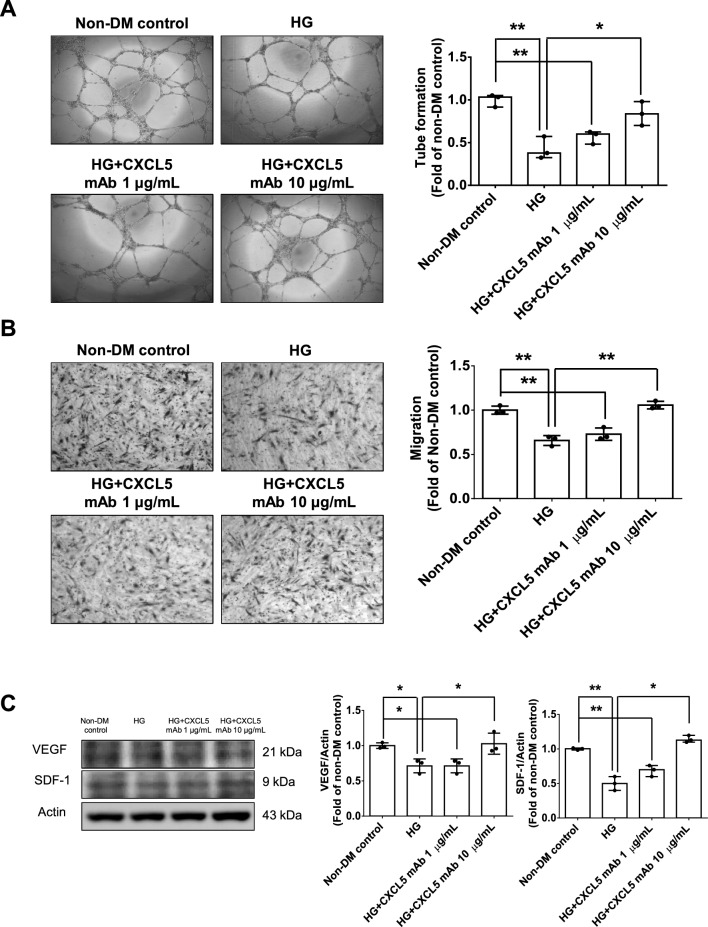

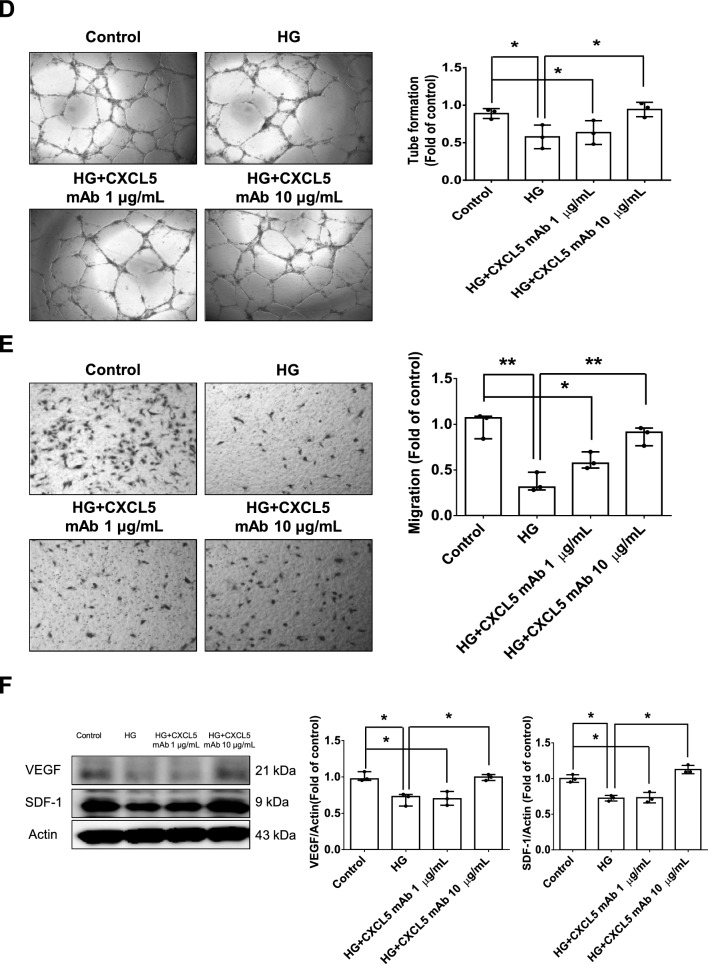
Treatment with CXCL5 neutralizing antibody upregulated VEGF/SDF-1 expression and promoted angiogenesis in late-EPCs from non-DM subjects and HAECs under the HG conditions. The network formation and migration abilities were improved after the administration of CXCL5 mAb in EPCs from non-DM subjects (n = 3; **A**, **B**). Western blotting and statistical analyses of VEGF and SDF-1 in EPCs from non-DM subjects (n = 3; **C**). The network formation and migration abilities were improved after the administration of CXCL5 mAb in HAECs (n = 3; **D**, **E**). Western blotting and statistical analyses of VEGF and SDF-1 in HAECs (n = 3; **F**). *CXCL5* C-X-C motif chemokine ligand 5, *EPC* endothelial progenitor cell, *HG* high glucose, *HAEC* human aortic endothelial cell, *mAb*,monoclonal antibody, *SDF-1* stromal cell-derived factor 1, *VEGF* vascular endothelial growth factor. N represents the number of independent experiments on different days and in different experimental runs. The Mann–Whitney U test was used to determine statistically significant differences. *p < 0.05, **p < 0.01

Treatment with CXCL5 neutralizing antibody at 10 µg/mL repaired the network formation and migration abilities of HG-stimulated EPCs from non-DM subjects (Fig. 2A, B). Administration of CXCL5 neutralizing antibody at 10 µg/mL doses increased the protein expression of VEGF and SDF-1 in HG-stimulated EPCs from non-DM subjects (Fig. 2C). On the other hand, treatments with CXCL5 neutralizing antibody at 10 µg/mL repaired the ability of in vitro angiogenesis and migration under the HG conditions (Fig. 2D, E). Meanwhile, administration of CXCL5 neutralizing antibody at 10 µg/mL increased the protein expression of VEGF and SDF-1 in HG-stimulated HAECs compared to the HG group (Fig. 2F). According to these results, treatment with CXCL5 neutralizing antibody increased VEGF and SDF-1 protein expression to repair the impaired migration and in vitro angiogenesis ability of EPCs and HAECs under the HG-stimulations.

#### CXCL5 caused endothelial cell damage and exerted pro-inflammatory and anti-angiogenic effects via ERK/p65 activation through CXCR2

**Fig. 3:**
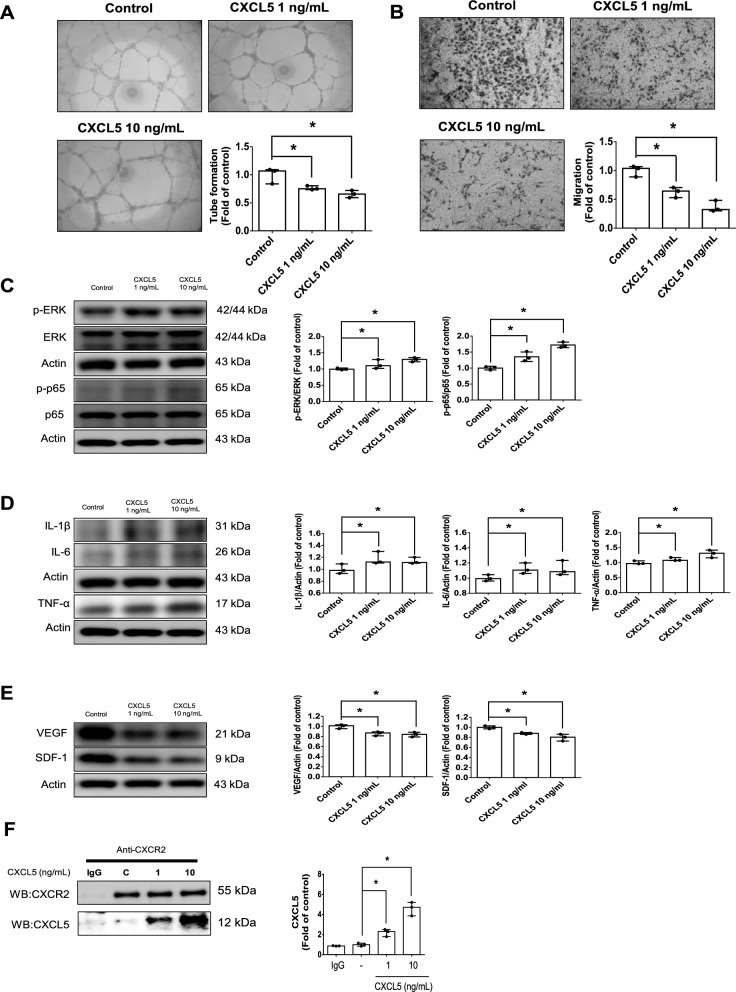
CXCL5 impaired vascular endothelial function via the ERK/p65 signaling pathway in HAECs. The network formation and migration abilities were impaired after administration of CXCL5 for 2 days (n = 3; **A**, **B**). Western blotting and statistical analyses of p-ERK, p-p65, IL-1β, IL-6, and TNF-α after administration of CXCL5 for 2 days (n = 3; **C**, **D**). Western blotting and statistical analyses of VEGF and SDF-1 after administration of CXCL5 for 2 days (n = 3; **E**). Western blotting of CXCR2 and CXCL5 after anti-goat IgG and CXCR2 immunoprecipitation (n = 3; **F**). *CXCL5* Chemokine C-X-C motif ligand 5, *CXCR2* Chemokine C-X-C motif receptor 2, *ERK* extracellular signal-regulated kinase, *HAEC* human aortic endothelial cell, *IL* interleukin, *SDF-1* stromal cell-derived factor 1, *TNF-α* tumor necrosis factor-α, *VEGF* vascular endothelial growth factor. N represents the number of independent experiments on different days and in different experimental runs. The Mann–Whitney U test was used to determine statistically significant differences. *p < 0.05, **p < 0.01

The administration of CXCL5 impaired network formation and migration abilities in HAECs (Fig. 3A, B). The phosphorylation of ERK and p65 were increased by the administration of CXCL5 in HAECs (Fig. 3C). Moreover, the downstream inflammatory protein including IL-1β, IL-6, and TNF-α were activation by CXCL5 treatments (Fig. 3D). On the other hand, the angiogenic protein expression such as VEGF and SDF-1 were reduced by CXCL5 treatments (Fig. 3E). The CXCL5-induced phosphorylation of p65 and its downstream inflammatory proteins including IL-1β, IL-6 and TNF-α were decreased by the administration of U0126, a ERK inhibitor, for 30 min and 4 h, respectively (Additional file 1: Fig. S2A). After administration of U0126 for 2 days, the decreased SDF-1 and VEGF expression by CXCL5 were reversed (Additional file 1: Fig. S2B).

The immunoprecipitation analysis showed that CXCL5 could interact with CXCR2 (Fig. 3F). The CXCL5-induced p-ERK, IL-1β, IL-6 and TNF-α were decreased by the administration of SB332235, a CXCR2 antagonist, for 4 h (Additional file 1: Fig. S2C). Furthermore, the decreased VEGF and SDF-1 expression by CXCL5 treatments were reversed after 2 days of SB332235 administration (Additional file 1: Fig. S2D). These results suggested that CXCL5 could impaired cell function and exerted pro-inflammation and anti-angiogenic effects via ERK/p65 activation through CXCR2.

### In vivo study

#### CXCL5 neutralizing antibody recovered neovasculogenesis in type 1 DM mice

**Fig. 4:**
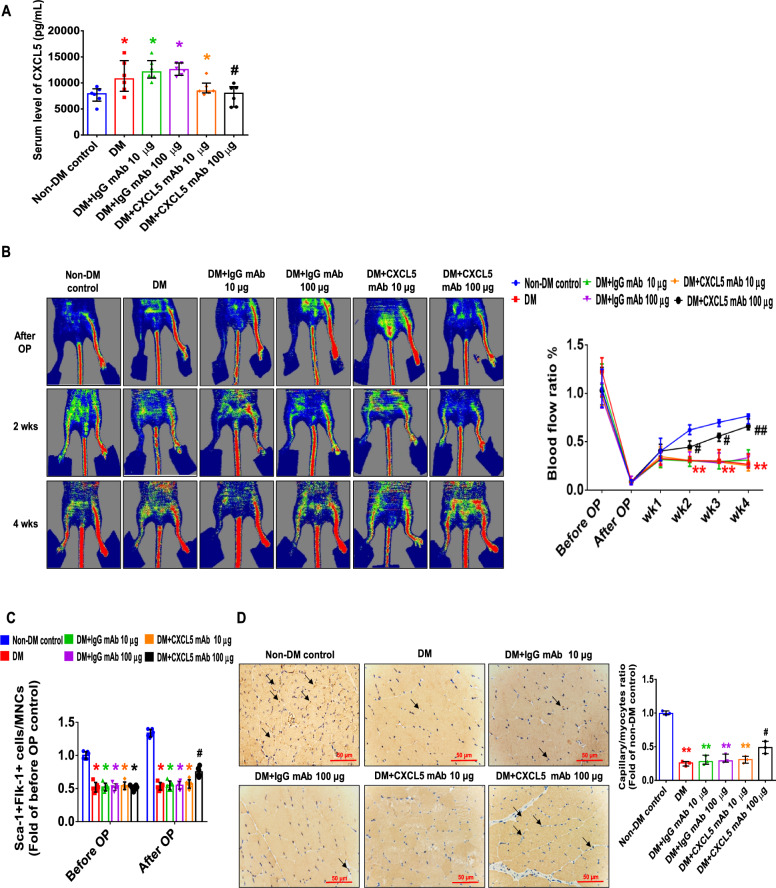

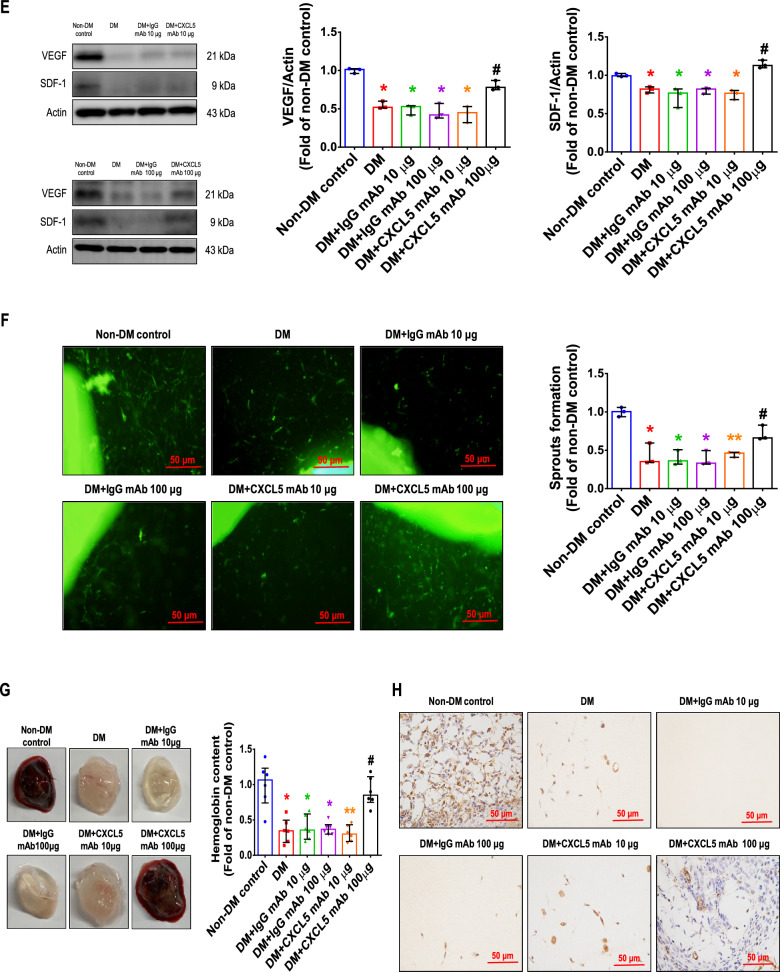

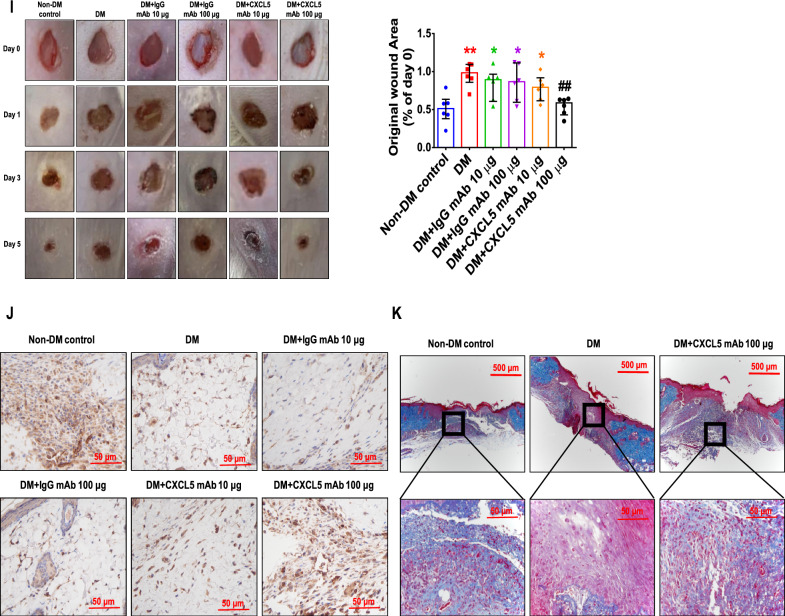
CXCL5 neutralizing antibody repaired neovascularization and wound healing in type 1 DM mice. Serum CXCL5 levels in the diabetic mice were higher than those in the non-DM control. Treatment with CXCL5 mAb reduced CXCL5 levels (n = 6; **A**). Representative evaluation of the ischemic (right) and nonischemic (left) hindlimbs before, immediately after 2 weeks and 4 weeks after the hindlimb ischemia surgery in STZ induced type 1 diabetic mice (n = 6; **B**). The number of circulating EPCs was determined by flow cytometry in STZ induced type 1 diabetic mice. Treatment with CXCL5 mAb 100 μg increased the number of circulating EPCs after ischemia surgery compared with DM (n = 6; **C**). Anti-CD31 immunostaining showed that CXCL5 mAb 100 μg treatment significantly increased the number of capillaries. Scale bar, 50 µm (n = 6; **D**) Western blotting and statistical analyses of VEGF and SDF-1 in the ischemia leg (n = 3; **E**). Angiogenesis in aortic ring cultures from CXCL5 mAb 100 μg mice was significantly increased the number of vessels sprouting compared with DM mice. Scale bar, 50 µm (n = 3; **F**). Representative matrigel plug images and analysis of hemoglobin content (n = 6; **G**). Representative matrigel plug images with immunostaining of CD31. CD31 positive areas were enhanced in the CXCL5 mAb 100 μg treatment mice. Scale bar, 50 µm (**H**). CXCL5 mAb 100 μg treatment improved wound repair ability in STZ induced type 1 diabetic mice. Representative wound areas and the closure rates of 3-mm punch biopsies were measured (n = 6; **I**). Representative wound area images with immunostaining of CD31. CD31 positive areas were enhanced in the CXCL5 100 μg mAb treatment mice. Scale bar, 50 µm (**J**). Representative skin images with masson trichrome staining. Collagen depositions were enhanced in the CXCL5 mAb 100 μg treatment mice. Scale bar, 50 and 500 µm (**K**). *CXCL5* Chemokine C-X-C motif ligand 5, *DM* diabetes mellitus, *EPC* endothelial progenitor cell, *mAb* monoclonal antibody, *STZ* Streptozotocin, *SDF-1* stromal cell-derived factor 1, *VEGF* vascular endothelial growth factor. The Mann–Whitney U test was used to determine statistically significant differences. *p < 0.05, **p < 0.01 compared with the non-DM control. ^#^p < 0.05, ^##^p < 0.01 compared with the untreated DM group

Diabetic mice had lower body weight and significantly higher blood glucose. There was no significant difference in body weight and blood sugar between the CXCL5 mAb-treated mice and the untreated diabetic ones (Additional file 1: Fig S3A, B). Serum CXCL5 levels were up-regulated in STZ-induced diabetic mice and were reduced in the CXCL5 mAb-treated mice (Fig. 4A). Blood flow in the ischemic hindlimb was equally reduced after hindlimb ischemia surgery in each group of mice. The blood flow was repaired by treatment with CXCL5 neutralizing antibody at 100 μg compared to the DM group (Fig. 4B). After hindlimb ischemia surgery, the number of EPCs in circulation was upregulated by treatment with CXCL5 neutralizing antibody in comparison to the DM and DM with IgG antibody treatment groups (Fig. 4C). In addition, the ischemic gastrocnemius muscle showed increased capillary density in the 100 μg CXCL5 neutralizing antibody-treated group (Fig. 4D). The protein expression of both VEGF and SDF-1 in the ischemic gastrocnemius muscle was increased by 100 μg CXCL5 neutralizing antibody after 4 weeks treatment compared to the DM and DM with IgG antibody treatment groups (Fig. 4E).

Moreover, more intricate in vivo study models such as aortic sprouting ring assay and matrigel plug assay were used to confirm the angiogenic impacts of CXCL5 inhibition in type 1 DM. The aortic ring assay had original vessel properties. However, aortic sprouting in aortic rings from the DM and IgG antibody treatment mice was overtly impaired. Administration of CXCL5 neutralizing antibody promoted vessel sprouting compared to the DM and IgG antibody treatment groups (Fig. 4F). In the matrigel plug assay, administration of CXCL5 neutralizing antibody at 100 μg enhanced hemoglobin content and capillary densities compared to the untreated DM group (Fig. 4G, H). Representative matrigel plug images with H&E staining were also shown (Additional file 1: Fig. S3C).

After a biopsy, cutaneous wound healing on the dorsal skin was demonstrated. The wound closure was essentially completed in the non-DM control group after 5 days. Treatment of CXCL5 neutralizing antibody at 100 μg increased the percentage of wound closure compared to the DM and DM with IgG antibody treatment groups after 3 days (Fig. 4I). Representative wound area images with H&E staining were also shown (Additional file 1: Fig. S3D). In addition, administration of CXCL5 neutralizing antibody increased CD31-positive vessel numbers and accumulated collagen deposition in the wound area compared to the DM group and IgG antibody treatment group (Fig. 4J, K). Altogether, strong evidence supported that CXCL5 inhibition could improve neovascularization in type 1 DM mice.

#### CXCL5 neutralizing antibody improved neovasculogenesis in type 2 DM mice

**Fig. 5:**
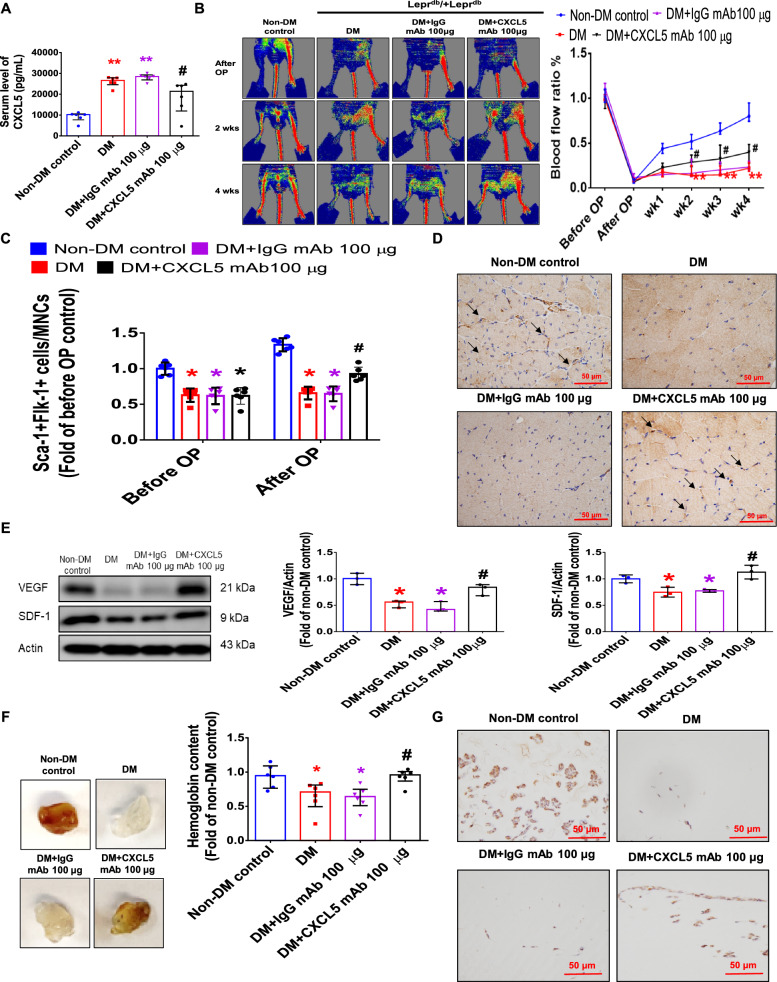

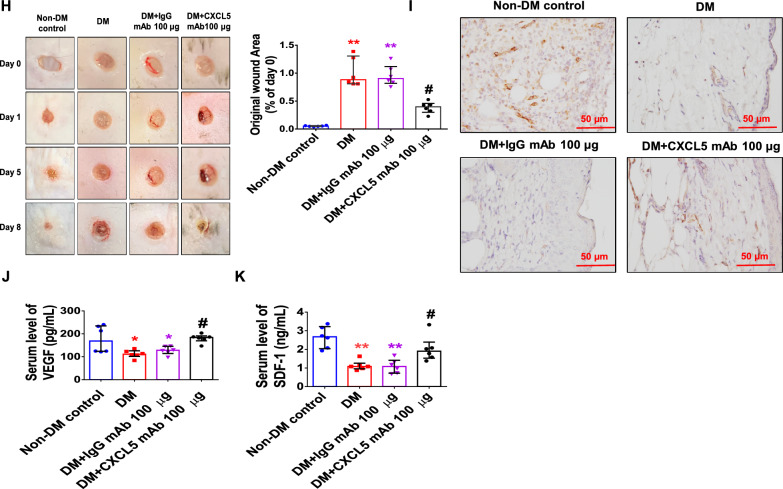
The neovascularization and wound healing were improved by neutralization of CXCL5 antibodies in type 2 DM mice. Treatment with CXCL5 mAb reduced CXCL5 levels in diabetic mice (n = 6; **A**). Representative evaluation of the ischemic (right) and nonischemic (left) hindlimbs before, immediately after 2 weeks and 4 weeks after the hindlimb ischemia surgery in db/db mice (n = 6; **B**). The number of circulating EPCs was determined by flow cytometry in db/db mice. Treatment with CXCL5 mAb 100 μg increased the number of circulating EPCs after ischemia surgery compared with DM (n = 6; **C**). Anti-CD31 immunostaining showed that CXCL5 mAb 100 μg treatment significantly increased the number of capillaries. Scale bar, 50 µm (n = 6; **D**). Western blotting and statistical analyses of VEGF and SDF-1 in the ischemia leg (n = 3; **E**). Representative matrigel plug images and analysis of hemoglobin content (n = 6; **F**). Representative matrigel plug images with immunostaining of CD31. CD31 positive areas were enhanced in the CXCL5 mAb 100 μg treatment mice. Scale bar, 50 µm (**G**). CXCL5 mAb 100 μg treatment improved wound repair ability in db/db mice. Representative wound areas and the closure rates of 3-mm punch biopsies were measured (n = 6; **H**). Representative wound area images with immunostaining of CD31. CD31 positive areas were enhanced in the CXCL5 mAb 100 μg treatment mice. Scale bar, 50 µm (**I**). Serum concentration of VEGF and SDF-1 were higher in the CXCL5 mAb 100 μg treatment mice (n = 6; **J**, **K**). *CXCL5* Chemokine C-X-C motif ligand 5, *DM* diabetes mellitus, *EPC* endothelial progenitor cell, *mAb* monoclonal antibody, *SDF-1* stromal cell-derived factor 1, *VEGF* vascular endothelial growth factor. The Mann–Whitney U test was used to determine statistically significant differences. *p < 0.05, **p < 0.01 compared with the non-DM control. ^#^p < 0.05, ^##^p < 0.01 compared with the untreated DM group

Diabetic mice had higher body weight and blood glucose levels. There was no significant difference in body weight and blood sugar between the CXCL5 mAb-treated mice and the untreated diabetic ones (Additional file 1: Fig. S4A, B). Serum CXCL5 levels were up regulated in diabetic mice and were reduced in the CXCL5 mAb-treated mice (Fig. 5A). After hindlimb ischemia surgery, the blood flow was repaired by treatment with CXCL5 neutralizing antibody compared to the DM group (Fig. 5B). The number of EPCs in circulation in non-DM mice was significantly increased after surgery. Nevertheless, the number of EPCs did not increase after surgery in the DM and IgG antibody groups but was reversed in the CXCL5 neutralizing antibody-treated group (Fig. 5C). In addition, capillary density was increased in the CXCL5 neutralizing antibody-treated group (Fig. 5D). The protein expression of both VEGF and SDF-1 in the ischemic gastrocnemius muscle was increased by 100 μg CXCL5 neutralizing antibody after 4 weeks treatment compared to the DM and DM with IgG antibody treatment groups (Fig. 5E). Furthermore, treatment with CXCL5 neutralizing antibody could enhance hemoglobin content and vessel formation in the matrigel plug assay (Fig. 5F, G). Representative matrigel plug images with H&E staining were also shown (Additional file 1: Fig. S4C).

In the wound healing assay, treatment of CXCL5 neutralizing antibody promoted wound closure compared to the DM and DM with IgG antibody treatment groups (Fig. 5H). Representative wound area images with H&E staining were also shown (Additional file 1: Fig. S4D). Treatment with CXCL5 neutralizing antibody increased vessel numbers in the wound area (Fig. 5I). Inhibition of CXCL5 enhanced VEGF and SDF-1 level of serum (Fig. 5J, K). Taken together, these observations suggested that CXCL5 inhibition could improve neovascularization in type 2 DM mice.

#### The recovery of blood flow after hindlimb ischemia and wound healing were increased in STZ-induced CXCL5KO diabetic mice

**Fig. 6:**
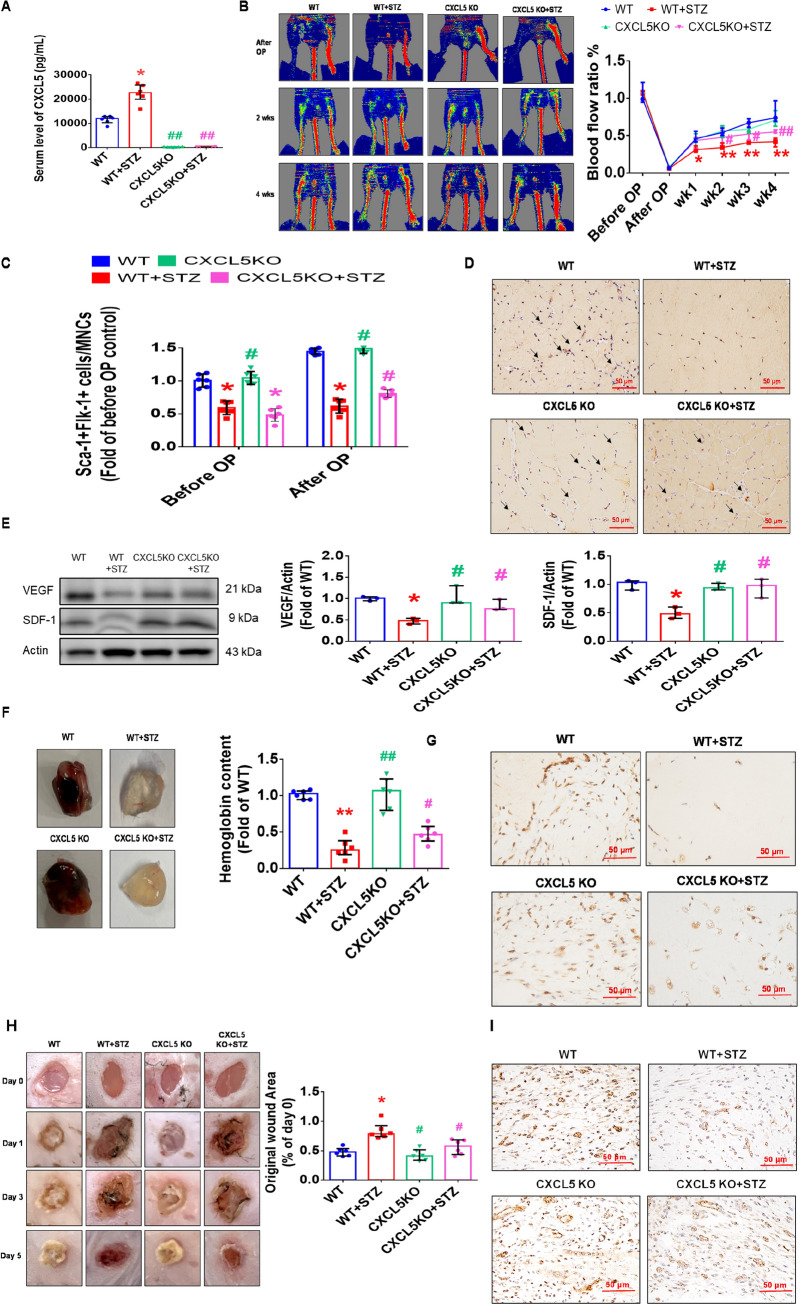
The neovascularization and wound healing were improved in STZ-induced CXCL5KO diabetic mice. CXCL5 knockout mice had rarely circulating CXCL5 (n = 6; **A**). Representative evaluation of the ischemic (right) and nonischemic (left) hindlimbs before, immediately after 2 weeks and 4 weeks after the hindlimb ischemia surgery in STZ induced type 1 diabetic mice (n = 6; **B**). The number of circulating EPCs was determined by flow cytometry in STZ induced type 1 diabetic mice. Deletion of CXCL5 expression increased the number of circulating EPCs after ischemia surgery compared with DM (n = 6; **C**). Anti-CD31 immunostaining showed that inhibition of CXCL5 expression significantly increased the number of capillaries. Scale bar, 50 µm (n = 6; **D**). Western blotting and statistical analyses of VEGF and SDF-1 in the ischemia leg (n = 3; **E**). Representative matrigel plug and analysis of hemoglobin content (n = 6; **F**). Representative matrigel plug images with immunostaining of CD31. CD31 positive areas were enhanced in the CXCL5 knockout diabetic mice. Scale bar, 50 µm (**G**). CXCL5 inhibition by knockout improved wound repair ability in STZ induced type 1 diabetic mice. Representative wound areas and the closure rates of 3-mm punch biopsies were measured (n = 6; **H**). Representative wound area images with immunostaining of CD31. CD31 positive areas were enhanced in the CXCL5 knockout mice. Scale bar, 50 µm (**I**). CXCL5, Chemokine C-X-C motif ligand 5; CXCL5KO, CXCL5-knockout mice; CXCL5KO+STZ, CXCL5 knockout diabetic mice. *DM* diabetes mellitus, *EPC* endothelial progenitor cell, *STZ* streptozotocin, *WT* wild-type mice, *WT+STZ* wild-type diabetic mice. The Mann–Whitney U test was used to determine statistically significant differences. *p < 0.05, **p < 0.01 compared with the WT group. ^#^p < 0.05, ^##^p < 0.01 compared with the WT+STZ group

Diabetic mice had lower body weight and significantly higher blood glucose. There was no significant difference in body weight and blood glucose between the CXCL5KO diabetic mice and the WT diabetic ones (Additional file 1: Fig. S5A, B). CXCL5 knockout mice had rarely circulating CXCL5 (Fig. 6A). To verify the role of CXCL5 in diabetic angiogenesis, STZ was used to induce type 1 DM in CXCL5KO mice. Four weeks after the hindlimb ischemia surgery, the STZ group had poor blood flow recovery. Importantly, the blood flow recovery was improved in the CXCL5KO+STZ group than the WT+STZ group (Fig. 6B). After the surgery, the number of EPCs in circulation was not increased in the WT+STZ group but was enhanced in the CXCL5+STZ group (Fig. 6C). CXCL5KO+STZ group showed higher capillary density in the ischemic gastrocnemius muscle compared to the WT+STZ group (Fig. 6D). The protein expression of both VEGF and SDF-1 in the ischemic gastrocnemius muscle was increased in CXCL5KO+STZ group after 4 weeks treatment compared to the WT+STZ groups (Fig. 6E). In the matrigel plug assay, CXCL5KO+STZ group also showed increased hemoglobin content and vessel formation in the matrigel plugs compared to the WT+STZ groups (Fig. 6F, G). Representative matrigel plug images with H&E staining were also shown (Additional file 1: Fig. S5C).

In the wound healing assay, the CXCL5KO+STZ group exerted increased percentage of wound closure compared to the WT+STZ group (Fig. 6H). Representative wound area images with H&E staining were also shown (Additional file 1: Fig. S5D). Furthermore, the CXCL5KO+STZ group had increased vessel numbers in the wound area (Fig. 6I). In summary, these findings indicated that CXCL5 deficiency could improve angiogenesis and neovascularization in DM.

## Discussion

**Fig. 7:**
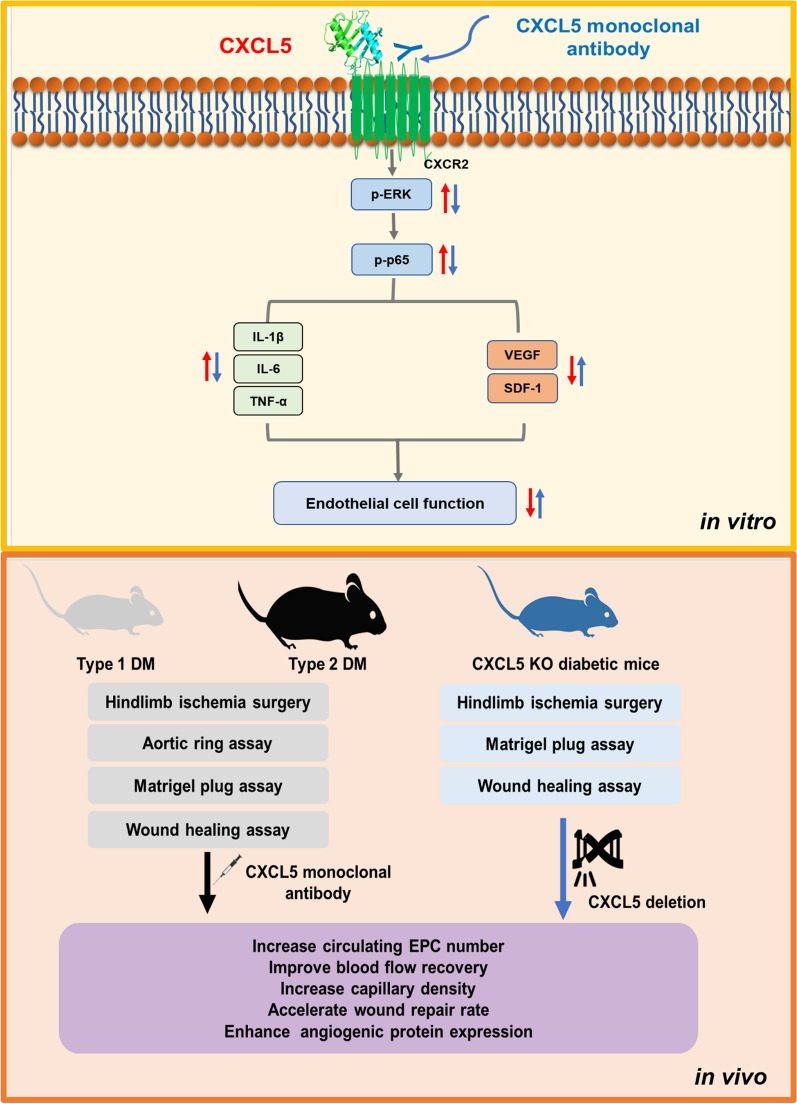
Summary of beneficial effects of CXCL5 suppression in diabetic vasculopathy. *CXCL5* Chemokine C-X-C motif ligand 5, *CXCR2* Chemokine C-X-C motif receptor 2, *EPC* endothelial progenitor cell, *ERK* extracellular signal-regulated kinase, *DM* diabetes mellitus, *IL* interleukin, *SDF-1* stromal cell-derived factor 1, *TNF-α* tumor necrosis factor-α, *VEGF* vascular endothelial growth factor

There were serial interesting and potentially important findings in this research. First, EPCs from type 2 DM patients released higher levels of CXCL5 than those from non-DM subjects. Inhibition of CXCL5 by neutralizing antibody improved EPC function from type 2 DM patients with enhanced VEGF and SDF-1 expression, and the results were the same in the HG-stimulated HAECs and EPCs from non-DM subjects. In addition, CXCL5 could directly induce endothelial cell damage and exert pro-inflammatory and anti-angiogenic effects via up-regulating ERK/p65 through CXCR2. Second, systemic CXCL5 suppression improved the blood flow recovery after hindlimb ischemia surgery, increased the number of EPCs in circulation, and upregulated VEGF and SDF-1 expressions in the ischemic gastrocnemius muscle in DM mice. Third, CXCL5 suppression not only increased hemoglobin content and vessel number in the matrigel plug assay but also enhanced the number of aortic spouting vessels in the aortic ring assay in DM mice. Fourth, CXCL5 suppression improved the wound closure area and wound area collagen accumulation in DM mice. Importantly, CXCL5KO mice were used to generate diabetic mice to further prove our concept. In line with the above observations, compared to common diabetic mice, CXCL5KO diabetic mice had improved angiogenesis and neovascularization in vivo. Taken together, our findings support the notion that CXCL5 may play a critical and mechanistic role in diabetic vasculopathy (Fig. 7).

These results were consistent with that of previous studies, which revealed significantly increased CXCL5 levels in a mouse model of type 2 DM and in patients with type 2 DM [15, 17, 18, 23, 24]. Macro- and microvasculopathy may develop with the progression of hyperglycemia in clinical settings [25]. EPCs have been well associated with vascular function and tissue regeneration. Upon tissue ischemia, EPCs can be mobilized from bone marrow for vascular repair and neovasculogenesis [26]. In type 2 DM patients, the number and function of EPCs are impaired with the presence of vascular complications [27]. On the other hand, neovascularization is driven by VEGF, which is necessary for endothelial proliferation, network formation, and migration. SDF-1 recruits EPCs from bone marrow and binds with CXCR4 to promote angiogenesis [28]. However, the VEGF and SDF-1 expressions are decreased in ischemic muscles and EPCs from DM patients [20]. In this study, we further showed that treatment with CXCL5 neutralizing antibody improved the cell function in EPCs from type 2 DM patients and in HG-stimulated EPCs from non-DM subjects as well as HAECs with upregulated VEGF and SDF-1 expressions in vitro. Meanwhile, CXCL5 inhibition repaired ischemia tissue angiogenesis with upregulated VEGF and SDF-1 in both mouse models of type 1 and type 2 DM in vivo.

While diabetic patients may develop PAD, the diabetic patients with PAD could have further impaired wound healing. Given that angiogenesis is essential for wound healing and tissue integrity, improving wound healing is one of the in vivo indicators of neovascularization. Previous in vivo study showed that diabetic mice had poorly healed wounds with elevated expressions of CXCL5 [29]. On the other hand, it was observed that decreased exudate CXCL5 level could be independently associated with the delayed wound healing in patients with diabetic foot [30]. However, it was not known if reducing serum CXCL5 could promote the development of diabetic foot ulcer and retard wound healing. While the mechanistic role of CXCL5 in diabetic foot ulcer healing seems controversial, further systemic investigation was required [30]. In our study, direct systemic inhibition on CXCL5 improved the wound healing with increased wound closure area as well as wound area collagen accumulation across different diabetic mouse models. The above findings were also confirmed in CXCL5 knockout diabetic mice. Accordingly, in line with our in vitro findings on EPCs from type 2 diabetic patients, our in vivo findings suggested that CXCL5 was not just an indicator, which might impair rather than promote angiogenesis as well as wound healing in diabetic animals. Given the potential role of CXCL5 in diabetic vasculopathy, future clinical study is indicated to elucidate if direct inhibition on systemic CXCL5 level may prevent the development of diabetic foot ulcer and improve wound healing in type 1 and type 2 diabetic patients.

There are some critical issues that may be further clarified. First, pro-inflammatory cytokines and chemokines may participate in the pathological role in the diabetes. Inflammation may lead to impaired neovascularization and cause dysfunctional EPCs and endothelial cells. We have previously showed that a pro-inflammatory chemokine-macrophage inflammatory protein (MIP)-1β is associated with diabetic neovascularization. MIP-1β reduced CXCR4 expression and caused dysfunctional EPCs, which impaired angiogenesis [20]. On the other hand, CXCL5 was shown to activate p65 and interleukin (IL)-1β promoter in rat cardiac-derived endothelial cells. In addition, neutrophils activated by CXCL5 amplified the inflammatory response, which promoted endothelial cell apoptosis [31]. Based on our results, it was first revealed that direct CXCL5 inhibition improved endothelial cell function and increased pro-angiogenic protein expression in vivo and in vitro. While both MIP-1β and CXCL5 and probably other chemokines may impair angiogenesis in diabetes, the specific in vivo anti-inflammatory effects via CXCL5 suppression can be further elucidated in future. Second, previous study indicated that CXCL5 inhibition by neutralizing antibody could improve insulin sensitivity and glucose clearance in insulin-resistant-obese mice [18, 19]. In the current study, the levels of fasting glucose were not significantly changed by direct inhibition or deficiency of CXCL5 in different diabetic animal models. The effects of CXCL5 inhibition on fasting blood glucose were different between the current and previous studies, which might be related to different dosages and duration of the antibody treatment as well as the different age and different models of the experimental animals (db/db type 2 diabetic mice and STZ-induced type 1 diabetic mice). In the current in vivo study, the pancreatic islet tissue, insulin levels, and glucose tolerance test were not checked. It is not known, though less likely, if the change of insulin level and insulin resistance may impact on the effects of CXCL5 inhibition on angiogenesis in the different animal models. However, the findings of our in vitro study did indicate the beneficial effects of direct CXCL5 inhibition on EPCs from type 2 diabetic patients and that on HG-treated EPCs from non-DM subjects. Given the major role of EPCs and endothelial cells in angiogenesis, our findings support the direct contribution of CXCL5 inhibition to improve angiogenesis and wound healing in in vivo DM and these beneficial effects might be resulting from its angiogenic and anti-inflammatory abilities. Third, our previous study showed that MIP-1β inhibition increased circulating EPCs by promoting their homing ability from bone marrow to the ischemic areas [20]. Here, we observed that CXCL5 inhibition increased numbers of circulating EPC, this might be due to the improved homing ability. However, other possibilities such as increased differentiation and proliferation or improved survival should be further addressed.

In conclusion, plasma CXCL5 was increased in type 2 diabetic patients. Direct CXCL5 suppression improved both EPCs and HAECs function in DM. CXCL5 could up-regulate IL-1β/IL-6/TNF-α and down-regulated VEGF/SDF-1 via ERK/p65 activation through CXCR2. Furthermore, the suppression of CXCL5 increased EPCs number in circulation, improved ischemic leg blood flow recovery, elevated the matrigel hemoglobin contents and vessel numbers, increased aortic ring neovascularization, and increased wound closure in different diabetic animal models. The similar findings could be also seen in CXCL5 knockout diabetic mice. Taken together, CXCL5 might contribute to vascular impairment in both clinical and experimental DM. A novel therapeutic strategy targeting on CXCL5 may be warranted to validate as the potential treatment for diabetic vascular complications in the future.

## Supplementary Information

**Additional file 1: Table S1.** Clinical characteristics of the study population. **Figure S1.** Morphology and characterization of human EPCs from peripheral blood. **Figure S2.** CXCL5 exerted pro-inflammatory and anti-angiogenic effects via ERK/p65 activation through CXCR2. **Figure S3.** The effects of CXCL5 neutralizing antibody on body weight, blood glucose, and H&E staining of section from matrigel plugs and wound areas in type 1 DM mice. **Figure S4.** The effects of CXCL5 neutralizing antibody on body weight, blood glucose, and H&E staining of section from matrigel plugs and wound areas in type 2 DM mice. **Figure S5.** The body weight, blood glucose, and H&E staining of section from matrigel plugs and wound areas in STZ-induced CXCL5KO diabetic mice. **Figure S6.** Complete Western blotting gels in this study.

## Data Availability

All data generated or analyzed during this study are included in this published article.

## Abbreviations

CXCL5: Chemokine C-X-C motif ligand 5
CXCL5KO: CXCL5 knockout
CXCR2: Chemokine C-X-C motif receptor 2
DM: Diabetes mellitus
EPCs: Endothelial progenitor cells
ERK: Extracellular signal-regulated kinase
HAECs: Human aortic endothelial cells
HG: High glucose
IL: Interleukin
PAD: Peripheral artery disease
SDF-1: Stromal cell-derived factor-1
STZ: Streptozotocin
TNF: Tumor necrosis factor
VEGF: Vascular endothelial growth factor
WT: Wild-type
