# Correction: CXCL5 suppression recovers neovascularization and accelerates wound healing in diabetes mellitus

**DOI:** 10.1186/s12933-023-01929-x

**Published:** 2023-08-01

**Authors:** Ching Chen, Liang-Yu Lin, Jaw-Wen Chen, Ting-Ting Chang

**Affiliations:** 1 Department and Institute of Pharmacology, National Yang Ming Chiao Tung University, Taipei, Taiwan; 2 School of Medicine, National Yang Ming Chiao Tung University, Taipei, Taiwan; 3 Division of Endocrinology and Metabolism, Department of Medicine, Taipei Veterans General Hospital, Taipei, Taiwan; 4 Healthcare and Services Center, Taipei Veterans General Hospital, Taipei, Taiwan; 5 Division of Cardiology, Department of Medicine, Taipei Medical University Hospital, Taipei, Taiwan; 6 Cardiovascular Research Center, National Yang Ming Chiao Tung University, Taipei, Taiwan; 7 Taipei Cardiovascular Research Center, Taipei Medical University, Taipei, Taiwan; 8 Biomedical Industry, National Yang Ming Chiao Tung University, Taipei, Taiwan

**Correction: Cardiovascular Diabetology (2023) 22:172** **https://doi.org/10.1186/s12933-023-01900-w**

Following publication of the original article [1], the author noticed an error in the images of Fig. 6H. The two images of Fig. 6H filled with red colours without any pictures which has been incorrectly processed by the typesetting team. This has now been corrected with this erratum (Fig. 6).

**Fig. 6:**
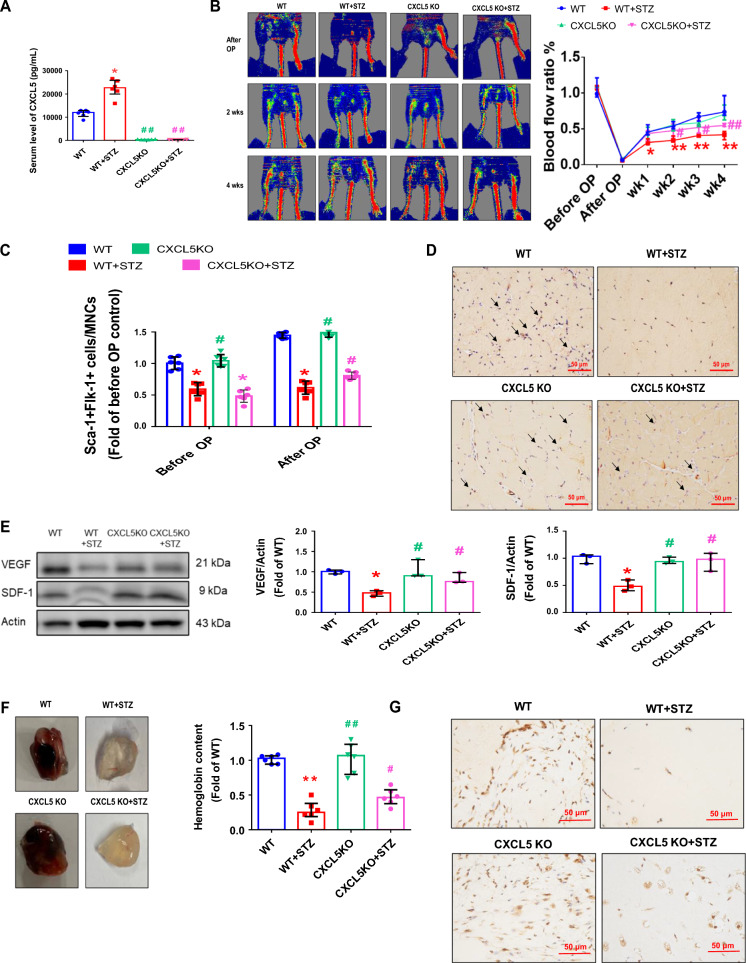

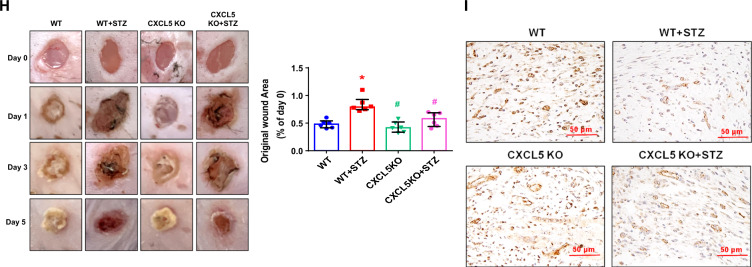
The neovascularization and wound healing were improved in STZ-induced CXCL5KO diabetic mice. CXCL5 knockout mice had rarely circulating CXCL5 (n = 6; **A**). Representative evaluation of the ischemic (right) and nonischemic (left) hindlimbs before, immediately after 2 weeks and 4 weeks after the hindlimb ischemia surgery in STZ induced type 1 diabetic mice (n = 6; **B**). The number of circulating EPCs was determined by flow cytometry in STZ induced type 1 diabetic mice. Deletion of CXCL5 expression increased the number of circulating EPCs after ischemia surgery compared with DM (n = 6; **C**). Anti-CD31 immunostaining showed that inhibition of CXCL5 expression significantly increased the number of capillaries. Scale bar, 50 µm (n = 6; **D**). Western blotting and statistical analyses of VEGF and SDF-1 in the ischemia leg (n = 3; **E**). Representative matrigel plug and analysis of hemoglobin content (n = 6; **F**). Representative matrigel plug images with immunostaining of CD31. CD31 positive areas were enhanced in the CXCL5 knockout diabetic mice. Scale bar, 50 µm (**G**). CXCL5 inhibition by knockout improved wound repair ability in STZ induced type 1 diabetic mice. Representative wound areas and the closure rates of 3-mm punch biopsies were measured (n = 6; **H**). Representative wound area images with immunostaining of CD31. CD31 positive areas were enhanced in the CXCL5 knockout mice. Scale bar, 50 µm (**I**). CXCL5, Chemokine C-X-C motif ligand 5; CXCL5KO, CXCL5-knockout mice; CXCL5KO + STZ, CXCL5 knockout diabetic mice. *DM* diabetes mellitus, *EPC* endothelial progenitor cell, *STZ* streptozotocin, *WT* wild-type mice, *WT* + *STZ* wild-type diabetic mice. The Mann–Whitney U test was used to determine statistically significant differences. *p < 0.05, **p < 0.01 compared with the WT group. ^#^p < 0.05, ^##^p < 0.01 compared with the WT + STZ group

The original article has been corrected.
