# Supplementary material for: CXCL5 suppression recovers neovascularization and accelerates wound healing in diabetes mellitus
Source: Cardiovasc Diabetol. 2023 Jul 7;22:172. doi: 10.1186/s12933-023-01900-w (PMC10329364; doi:10.1186/s12933-023-01900-w)
Supplement: Supplementary file 1 — Additional file 1: Table S1. Clinical characteristics of the study population. Figure S1. Morphology and characterization of human EPCs from peripheral blood. Figure S2. CXCL5 exerted pro-inflammatory and anti-angiogenic effects via ERK/p65 activation through CXCR2. Figure S3. The effects of CXCL5 neutralizing antibody on body weight, blood glucose, and H&E staining of section from matrigel plugs and wound areas in type 1 DM mice. Figure S4. The effects of CXCL5 neutralizing antibody on body weight, blood glucose, and H&E staining of section from matrigel plugs and wound areas in type 2 DM mice. Figure S5. The body weight, blood glucose, and H&E staining of section from matrigel plugs and wound areas in STZ-induced CXCL5KO diabetic mice. Figure S6. Complete Western blotting gels in this study. [file 12933_2023_1900_MOESM1_ESM.pdf]

Supplemental materials

CXCL5 suppression recovers neovascularization and accelerates wound healing in diabetes mellitus

Ching Chen, et al.

Table S1

	Non-diabetic subjects (n=6)		Type 2 DM patients (n=6)	
	Median	Q1-Q3	Median	Q1-Q3
Male gender (%)	50%		50%	
Age (years)	65	62-69	64	61-70
Fasting glucose (mg/dL)	103	101-103	124 **	121-142
HbA1c (%)	6	5-6	8 **	7-8
Cholesterol (mg/dL)	177	166-206	160	150-173
Triglycerides (mg/dL)	108	88-128	153	108-199
ACR (mg/g)	16.3	6-23	82	64-110
eGFR (ml/min/1.73m^2)	80	78-83	67	52-98
ALT (U/L)	36	34-41	37	34-43
AST (U/L)	37	33-43	39	33-38
Oral glucose-lowering drugs	-		100%	
Antihypertensive drugs	50%		67%	
Lipid-lowering drugs	33%		50%	

Table S1. Clinical characteristics of the study population. Data are presented as median with interquartile range. ACR, Albumin to creatinine ratio; ALT, Alanine aminotransferase; AST, Aspartate aminotransferase; DM, Diabetes mellitus; eGFR, Estimated glomerular filtration rate; HbA1c, Glycated hemoglobin. Data sets were analyzed with the non-parametric tests followed by a Mann-Whitney U test. ** $p<0.01$.

Figure S1

Endothelial Progenitor Cells (EPCs)

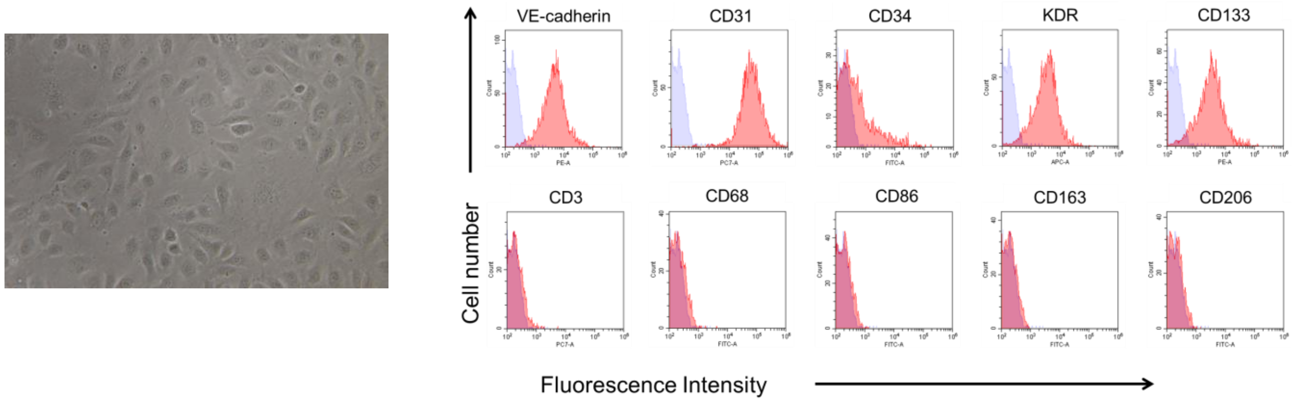

Figure S1. Morphology and characterization of human EPCs from peripheral blood. EPCs expressed endothelial-specific antigens, such as VE-cadherin, CD31, CD34, KDR, and CD133, and negatively expressed macrophage-specific antigen, such as CD3, CD68, CD86, CD163, and CD206.

Figure S2

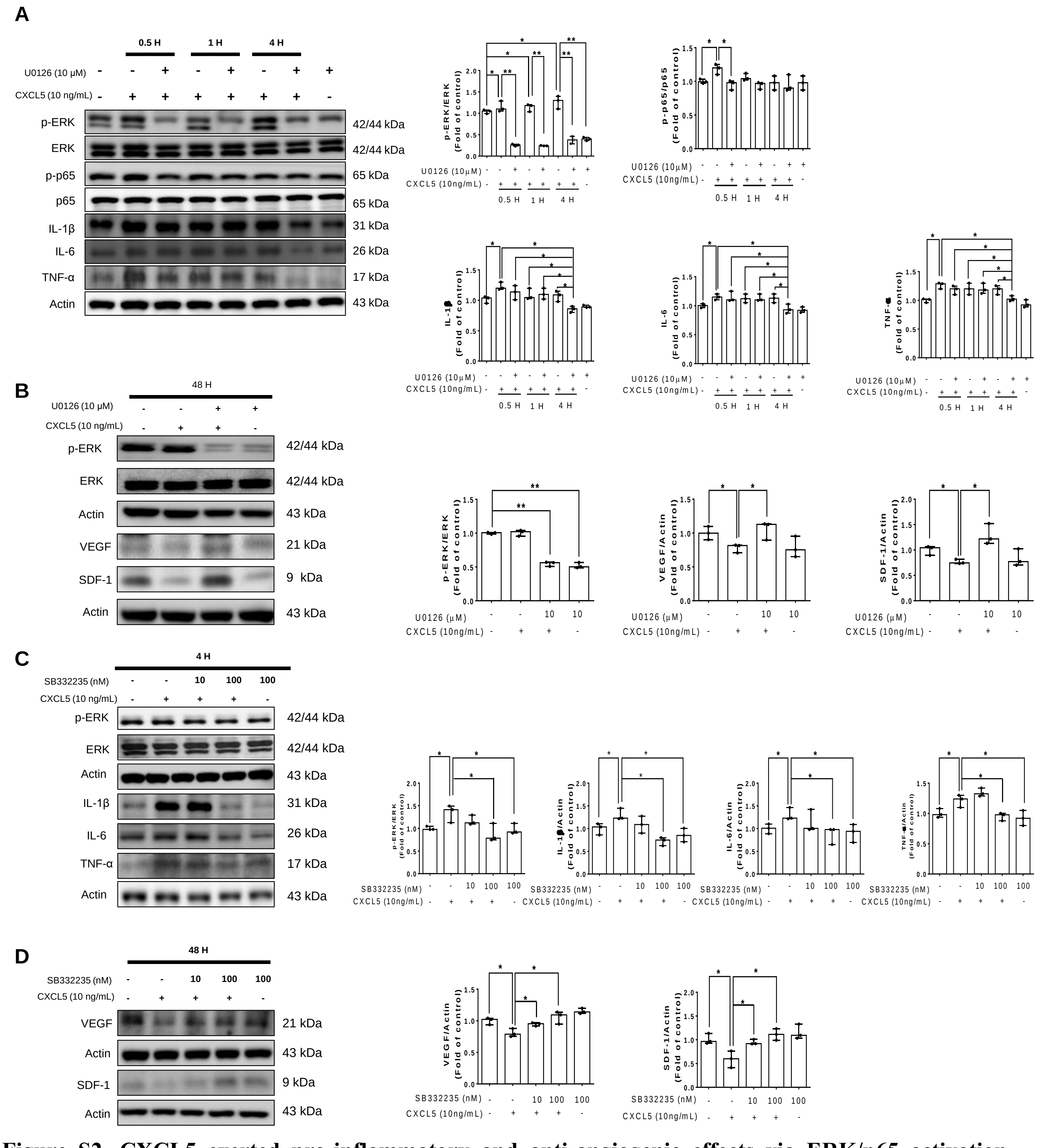

Figure S2. CXCL5 exerted pro-inflammatory and anti-angiogenic effects via ERK/p65 activation through CXCR2. Western blotting and statistical analyses of p-ERK, p-p65, IL-1β, IL-6 and TNF-α after administration of U0126 (n=3; A). Western blotting and statistical analyses of VEGF and SDF-1 after administration of U0126 (n=3; B). Western blotting and statistical analyses of p-ERK, IL-1β, IL-6 and TNF-α after administration of SB332235 (n=3; C) Western blotting and statistical analyses of VEGF and SDF-1 after administration of SB332235 (n=3; D). N represents the number of independent experiments on different days and in different experimental runs. Data sets were analyzed with the non-parametric tests followed by a Mann-Whitney U test. * $p<0.05$, ** $p<0.01$.

Figure S3

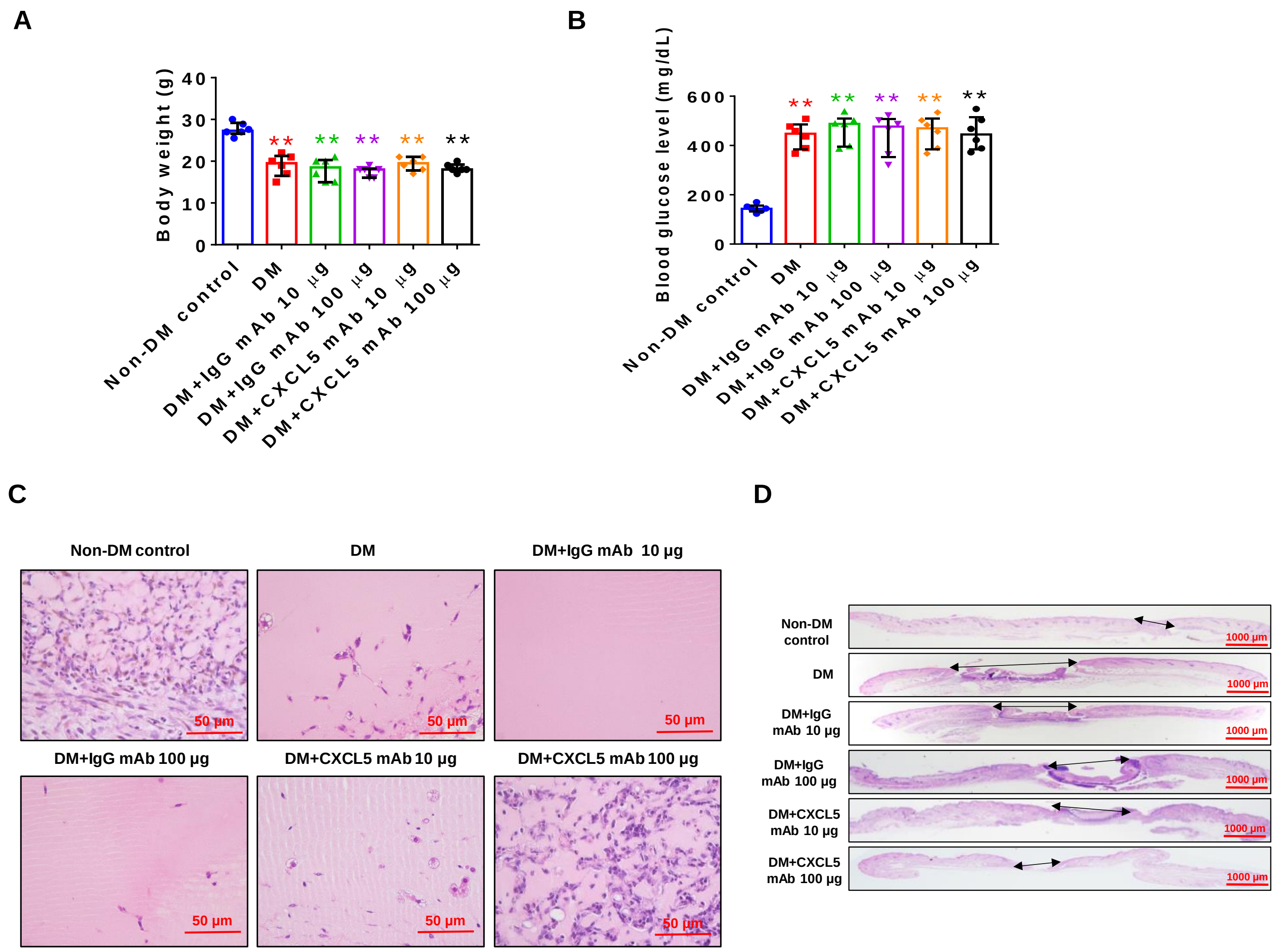

Figure S3. The effects of CXCL5 neutralizing antibody on body weight, blood glucose, and H&E staining of section from matrigel plugs and wound areas in type 1 DM mice. The body weights and blood glucose levels (n = 6; A and B). Representative matrigel plug images with H&E staining. Scale bar, 50 µm (C). Representative wound area images with H&E staining. Scale bar, 1000 µm (D). Data sets were analyzed with the non-parametric tests followed by a Mann-Whitney U test. ** p<0.01.

Figure S4

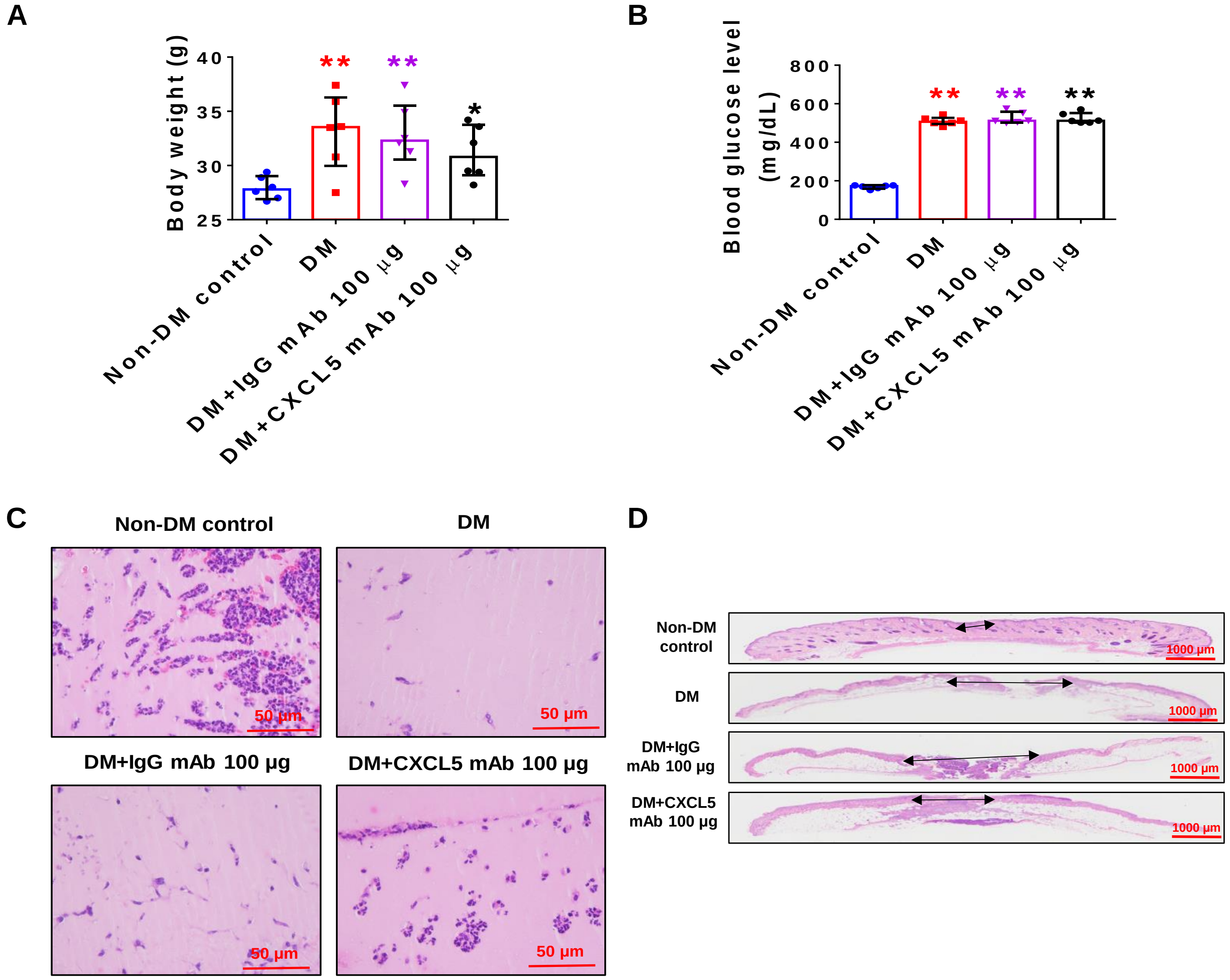

Figure S4. The effects of CXCL5 neutralizing antibody on body weight, blood glucose, and H&E staining of section from matrigel plugs and wound areas in type 2 DM mice. The body weights and blood glucose levels (n = 6; A and B). Representative matrigel plug images with H&E staining. Scale bar, 50 μm (C). Representative wound area images with H&E staining. Scale bar, 1000 μm (D). Data sets were analyzed with the non-parametric tests followed by a Mann-Whitney U test. * $p<0.05$, ** $p<0.01$.

Figure S5

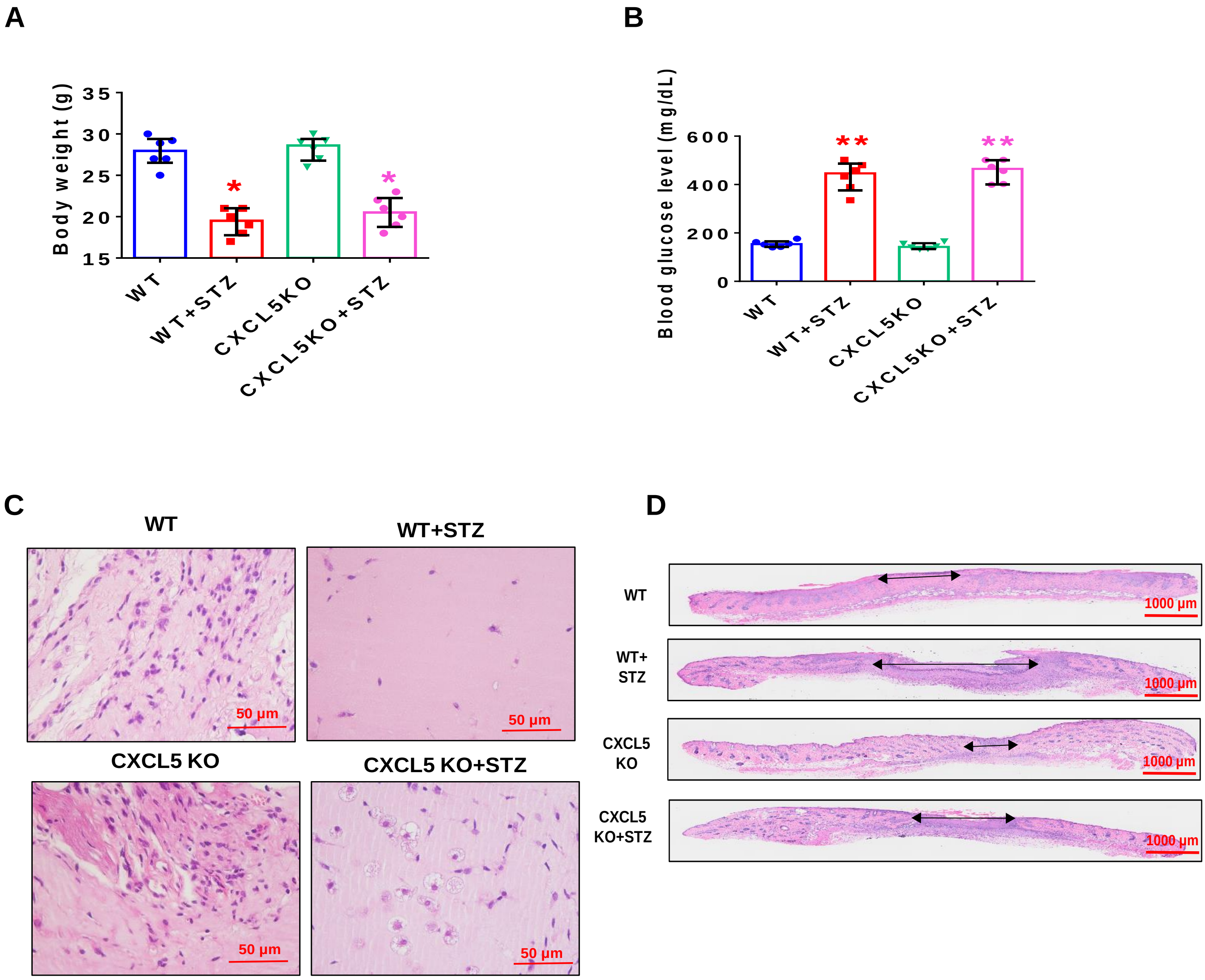

Figure S5. The body weight, blood glucose, and H&E staining of section from matrigel plugs and wound areas in STZ-induced CXCL5KO diabetic mice. The body weights and blood glucose levels (n = 6; A and B). Representative matrigel plug images with H&E staining. Scale bar, 50 μm (C). Representative wound area images with H&E staining. Scale bar, 1000 μm (D). Data sets were analyzed with the non-parametric tests followed by a Mann-Whitney U test. * $p<0.05$, ** $p<0.01$.

Figure S6

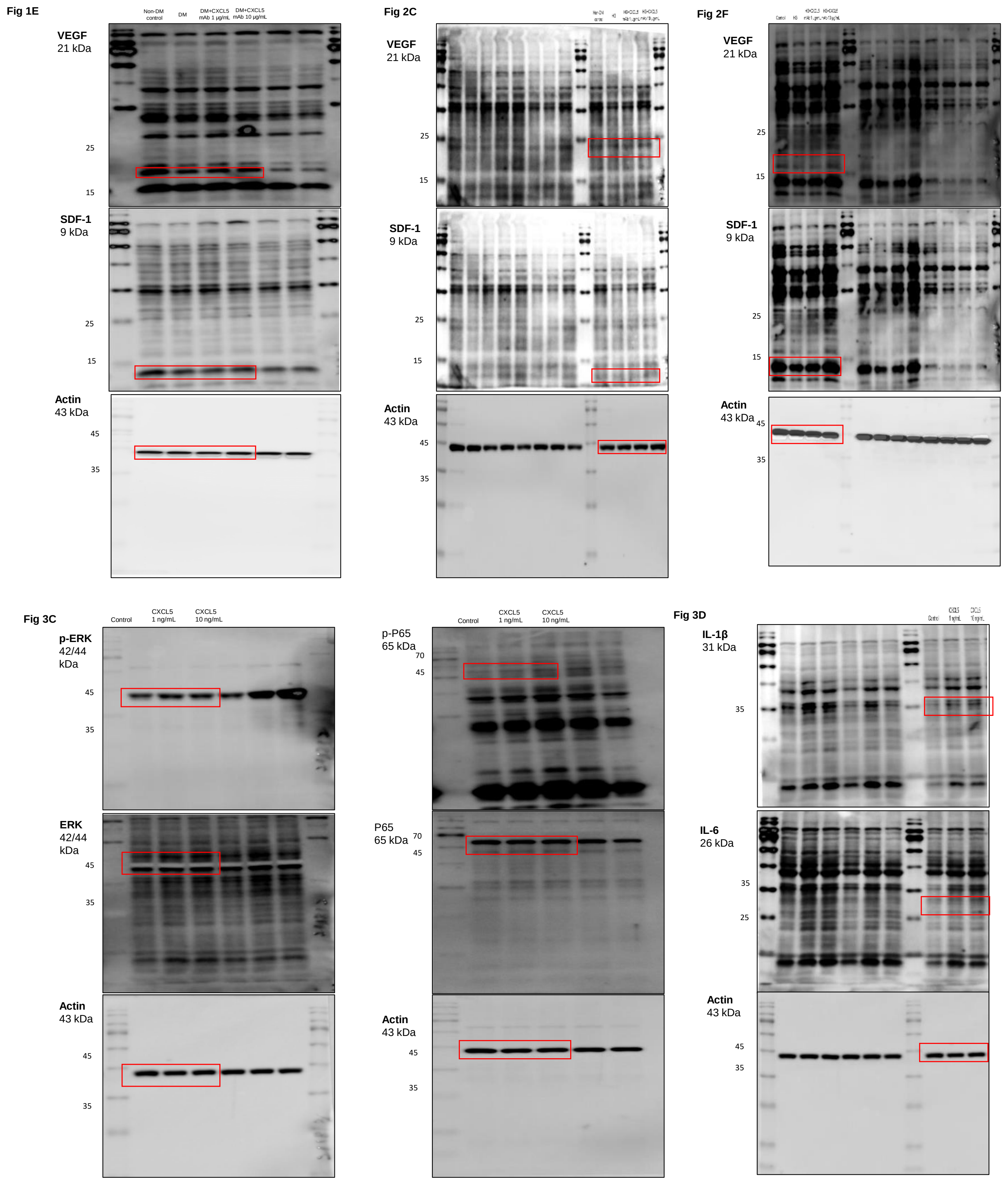

Fig 1E
Non-DM control
DM
DM+CXCL5 mAb 1 μg/mL
DM+CXCL5 mAb 10 μg/mL
VEGF
21 kDa
25
15
SDF-1
9 kDa
25
15
Actin
43 kDa
45
35
Fig 2C
VEGF
21 kDa
25
15
SDF-1
9 kDa
25
15
Actin
43 kDa
45
35
Fig 2F
VEGF
21 kDa
25
15
SDF-1
9 kDa
25
15
Actin
43 kDa
45
35
Fig 3C
Control
CXCL5 1 ng/mL
CXCL5 10 ng/mL
p-ERK
42/44 kDa
45
35
ERK
42/44 kDa
45
35
Actin
43 kDa
45
35
Control
CXCL5 1 ng/mL
CXCL5 10 ng/mL
p-P65
65 kDa
70
45
P65
65 kDa
70
45
Actin
43 kDa
45
35
Fig 3D
IL-1β
31 kDa
35
IL-6
26 kDa
35
25
Actin
43 kDa
45
35

Fig 3D

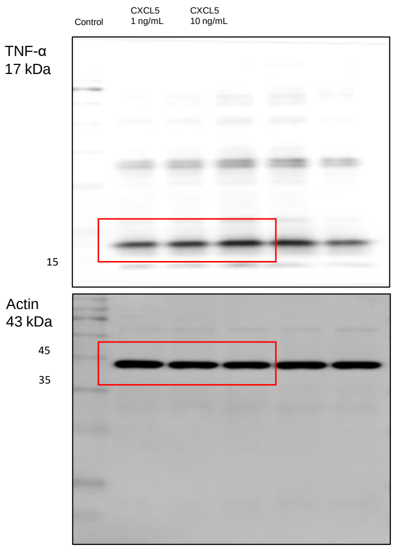

Fig 3E

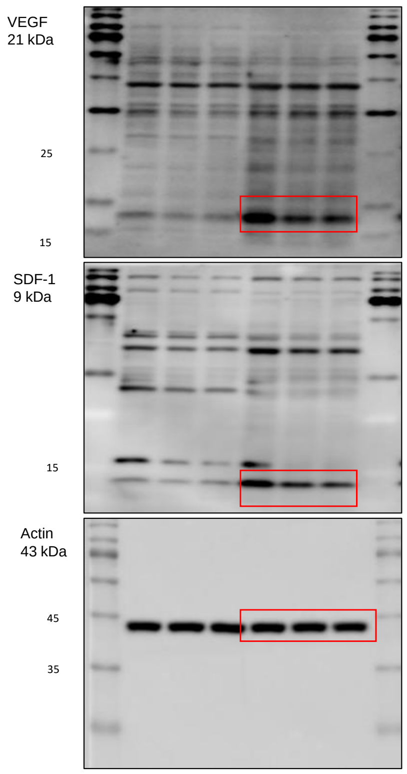

Fig 3F

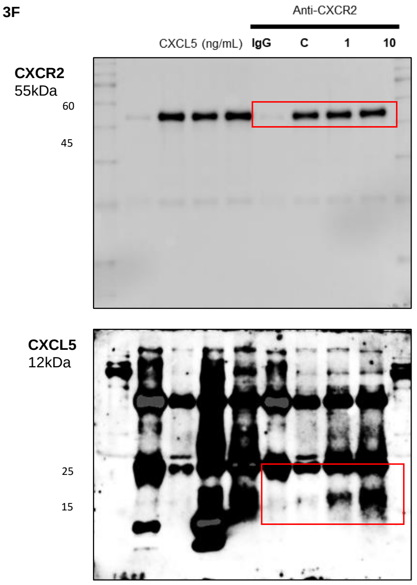

Fig 4E

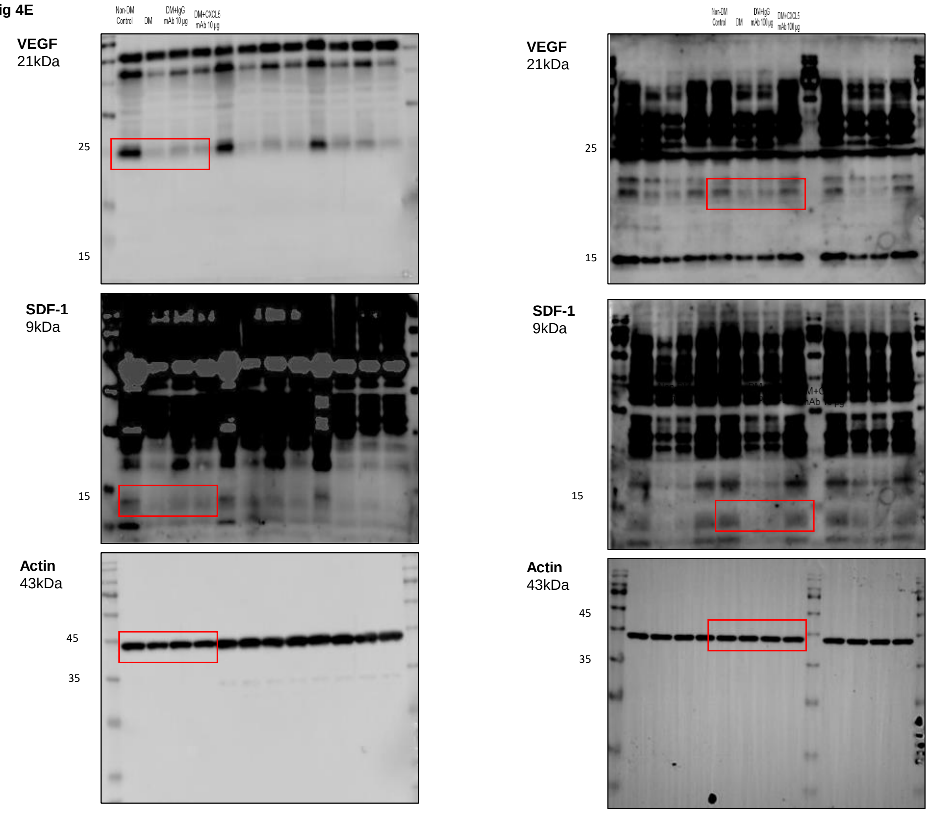

Fig 5E

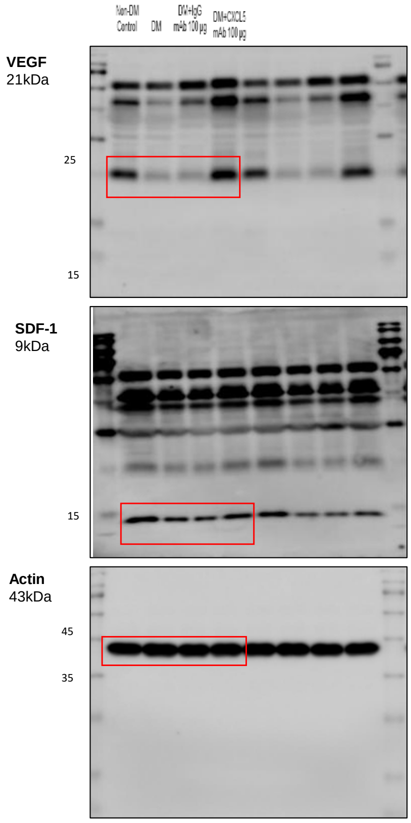

Fig 6E

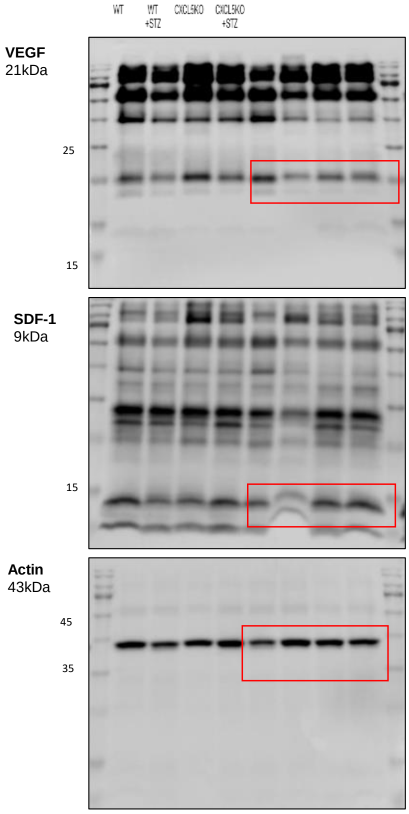

Fig 2SA

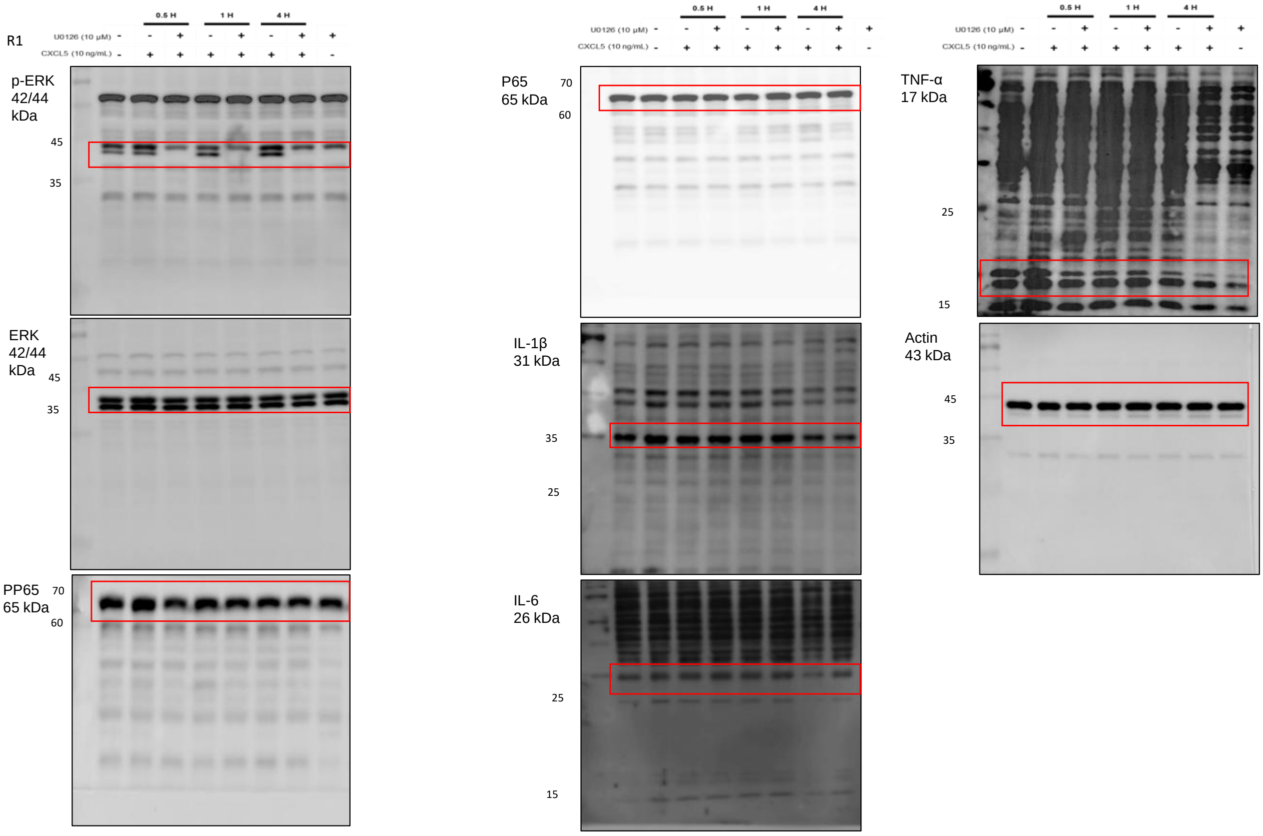

Fig 2SB

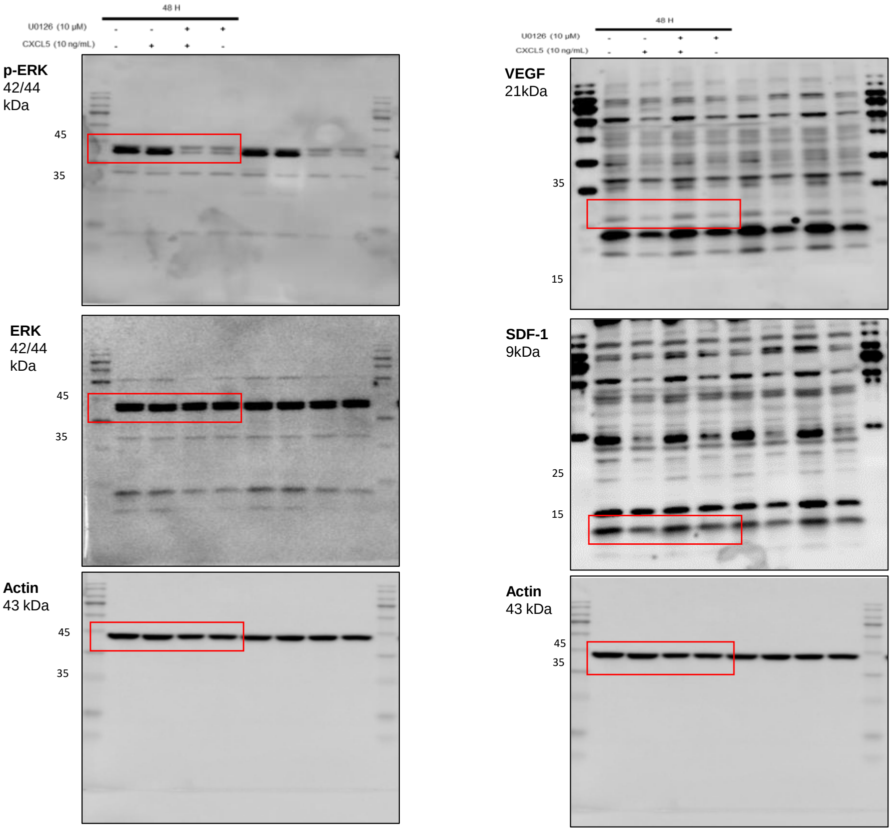

Fig 2SC

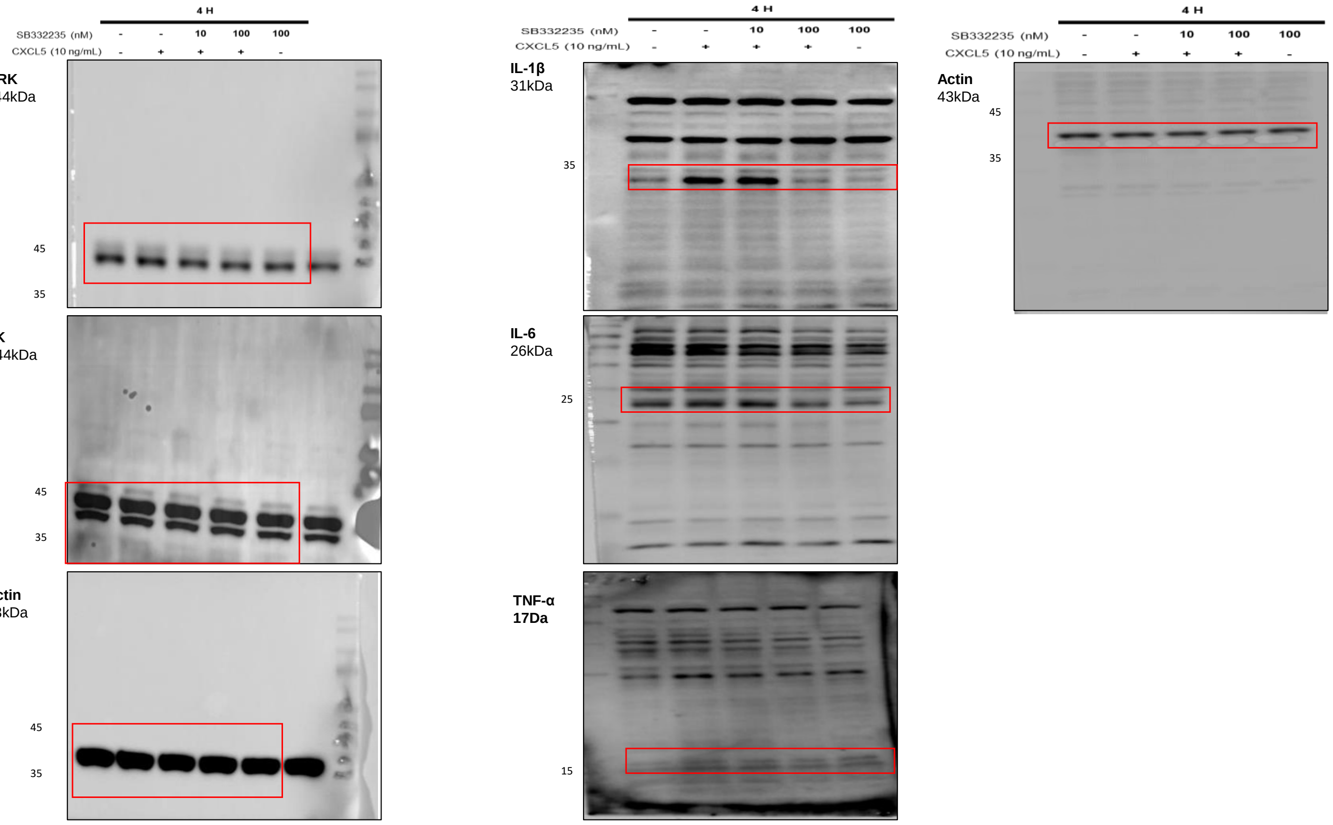

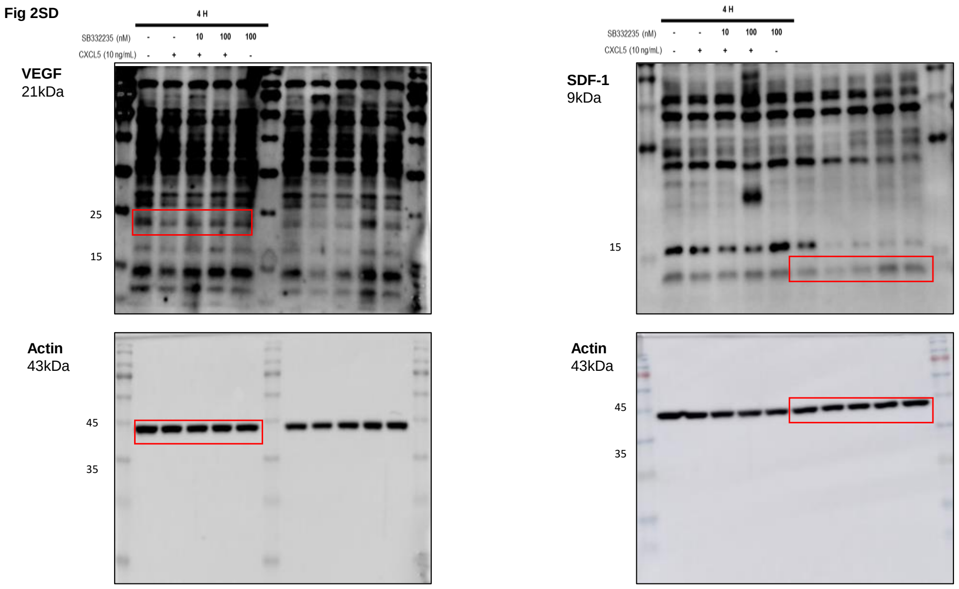

Figure S6. Complete Western blotting gels in this study.
